# Person centred care provision and care planning in chronic kidney disease: which outcomes matter? A systematic review and thematic synthesis of qualitative studies: Care planning in CKD: which outcomes matter?

**DOI:** 10.1186/s12882-021-02489-6

**Published:** 2021-09-13

**Authors:** Ype de Jong, Esmee M. van der Willik, Jet Milders, Yvette Meuleman, Rachael L. Morton, Friedo W. Dekker, Merel van Diepen

**Affiliations:** 1 Department of Clinical Epidemiology, Leiden University Medical Centre, Albinusdreef 2, 2333 ZA Leiden, The Netherlands; 2 Department of Internal Medicine, Leiden University Medical Centre, Leiden, The Netherlands; 3 NHMRC Clinical Trials Centre, Faculty of Medicine and Health, The University of Sydney, Sydney, Australia

## Abstract

**Rationale & Objective:**

Explore priorities related to outcomes and barriers of adults with chronic kidney disease (CKD) regarding person centred care and care planning.

**Study design:**

Systematic review of qualitative studies.

**Search Strategy & Sources:**

In July 2018 six bibliographic databases, and reference lists of included articles were searched for qualitative studies that included adults with CKD stages 1–5, not on dialysis or conservative management, without a previous kidney transplantation.

**Analytical Approach:**

Three independent reviewers extracted and inductively coded data using thematic synthesis. Reporting quality was assessed using the COREQ and the review reported according to PRISMA and ENTREQ statements.

**Results:**

Forty-six studies involving 1493 participants were eligible. The period after diagnosis of CKD is characterized by feelings of uncertainty, social isolation, financial burden, resentment and fear of the unknown. Patients show interest in ways to return to normality and remain in control of their health in order to avoid further deterioration of kidney function. However, necessary information is often unavailable or incomprehensible. Although patients and healthcare professionals share the predominant interest of whether or not dialysis or transplantation is necessary, patients value many more outcomes that are often unrecognized by their healthcare professionals. We identified 4 themes with 6 subthemes that summarize these findings: ‘pursuing normality and control’ (‘pursuing normality’; ‘a search for knowledge’); ‘prioritizing outcomes’ (‘reaching kidney failure’; ‘experienced health’; ‘social life’; ‘work and economic productivity’); ‘predicting the future’; and ‘realising what matters’. Reporting quality was moderate for most included studies.

**Limitations:**

Exclusion of non-English articles.

**Conclusions:**

The realisation that patients’ priorities do not match those of the healthcare professionals, in combination with the prognostic ambiguity, confirms fatalistic perceptions of not being in control when living with CKD. These insights may contribute to greater understanding of patients’ perspectives and a more person-centred approach in healthcare prioritization and care planning within CKD care.

**Supplementary Information:**

The online version contains supplementary material available at 10.1186/s12882-021-02489-6.

## Introduction

Chronic kidney disease (CKD) is a group of kidney diseases in which there usually is a gradual decrease in kidney function leading to kidney failure. The often asymptomatic nature of CKD, combined with the low awareness of kidney function in general [1], makes it difficult for patients to comprehend, cope and finally take control after the diagnosis of CKD [2–12]. During the progression of CKD to kidney failure however, numerous physical and psychosocial symptoms may develop, overall reducing health-related quality of life (HRQOL) [13, 14]. In this phase, kidney replacement therapy (KRT; kidney transplantation or dialysis), or alternatively conservative therapy is necessary, requiring an informed decision with knowledge of the disease, the possible outcomes and the chances of reaching these outcomes in combination with prioritization of what matters to the patient.

However, for most patients the period between CKD and kidney failure is marked by confusion about the disruptive transition from their pre- to their postdiagnosis self, and uncertainty about what to expect [10, 15, 16]. Furthermore, it is increasingly acknowledged that outcomes prioritized by clinicians, such as planning for dialysis or transplant, and postponing kidney failure and death, do not adequately reflect patients’ desired outcomes, which in contrast may include patient reported outcomes (PROs) like HRQOL or symptom burden [9, 17, 18]. PRO measures (PROMs) have been developed to further implement person-centred care, by providing insight into outcomes and enhancing the patient-professional conversation about patients’ needs and expectations. Such aspects of person-centred care show promising results but have yet to be fully incorporated into routine nephrological care [9, 19–21].

In-depth knowledge about what matters to patients can also be obtained through qualitative research. Moreover, by using qualitative methods, answers to why patients value these outcomes can also be obtained, hereby providing an opportunity for deeper understanding of their motivations, behaviour and beliefs. Though frequently used as a first step for the development of PROMs, transferability to other populations than the study subjects of single qualitative studies remains a concern. Systematically reviewing and thematically synthesizing the data of these single studies can result in a greater conceptual understanding of the topic beyond the single studies [22].

Although person-centred care within CKD shows promising results [23–25], better understanding of patients’ perspectives on what is important in nephrological care may help to further implement person-centred care. Hence, the aim of this study is to identify outcomes prioritized by patients with CKD, and barriers to person-centred care and care planning, by means of a systematic review and inductive thematic synthesis of qualitative studies among patients with CKD.

## Method

We followed the ENTREQ (Enhancing transparency in reporting the synthesis of qualitative research) [26] checklist and the PRISMA [27] statements for reporting our qualitative thematic synthesis.

### Selection criteria

All types of written qualitative studies in patients with CKD were included, where data had been collected via interviews, focus groups, or observations. Non-English articles were excluded to prevent cultural and linguistic bias in translation: the original meaning and interpretation may be lost in translation [28]. No publication date constraints were applied. We aimed to identify the priorities regarding outcomes and processes of care and barriers regarding person centred care of patients with CKD, not receiving KRT or conservative management and without a previous kidney transplant. Therefore, studies with mixed populations or mixed methods were excluded if the qualitative data related to patients with CKD was not presented separately. We excluded studies on children (< 18 years of age) because of different implications in shared decision making.

### Search methods and study selection

Systematically searching for qualitative studies aiming to identify all available studies, instead of a representative sample is problematic, as most bibliographic databases have different – if any – methods to identify qualitative research [29]. Building upon previous studies [29–31], we developed and piloted a comprehensive search method to identify all articles relevant to our subject, by including not only medical but also psychological bibliographic databases. We combined synonyms of “CKD” with synonyms for “qualitative”, “interview”, “focus group”, “perception”, “coping”, “barrier”, “prognosis”, and “preference” to develop search strings for PubMed, Embase, Web of Science, the Cochrane Library, PsycINFO and Emcare. After removal of duplicates, YdJ and EvdW independently selected the relevant titles, abstracts and full-text articles. Review articles and included original articles were checked for missing references (i.e. lateral- or cross-reference searching). Disagreements were resolved by discussion with MvD and YM. Information on the pilot search, the detailed search method, and overview of the search strings and study selection is given in the supplement (Supplementary Item 1 and Fig. S1).

### Data extraction, quality assessment and synthesis of findings

Data on CKD stage, patient demographics and study methodology were independently extracted by YdJ, EvdW and JM. Correctness of extracting and the accuracy of study characteristics requiring interpretation (e.g. study methodology if not stated by the author) were checked by YdJ and EvdW. Disagreements were resolved by discussion. The COnsolidated criteria for REporting Qualitative research (COREQ), a 32-item checklist [32], was used to assess reporting quality. In line with previous studies [33, 34], we categorized studies as having good (≥25 items); moderate (17–24 items); poor (9–16 items); or very poor (≤8 items) reporting quality. A systematic approach following the standards for systematic reviews of qualitative research, as established by the Cochrane Collaboration [33], was used and adapted to our study design. We grouped the included articles in two groups: 1) studies including only patients with CKD, and 2) studies including patients with CKD and other participants, but with sections identifiable as data from patients with CKD. For the first group, all text under ‘results’ and ‘discussion/conclusion’ section was used in the analysis; for the second group, only data that could be linked to patients with CKD was extracted. Transcripts were analysed thematically [34, 35]. Articles were read multiple times to familiarize ourselves with the data, and line by line coding of the designated parts was conducted by YdJ, EvdW and JM, summarizing the data using both descriptive and interpretative approaches. Then, by clustering the codes, descriptive themes were identified inductively from the data. As our analysis was inductive, we did not use a predefined or existing coding frame, but developed our own coding frame fitting the data. Coding and analysis was conducted by YdJ, EvdW and JM independently. Hereafter, a discussion on the meaning of the coded text followed in which the coding of the themes was uniformized and the coding tree expanded. After agreement on the coding tree, main themes were created by constant comparison, grouping similar subthemes and organizing subthemes hierarchically into meaningful main themes. To judge consistency of interpretation, themes were compared and discussed. We included and coded all eligible studies. ATLAS.ti software (GmbH, Berlin, version 7.5.18) was used for the coding process.

## Results

### Literature search and patient characteristics

**Fig. 1:**
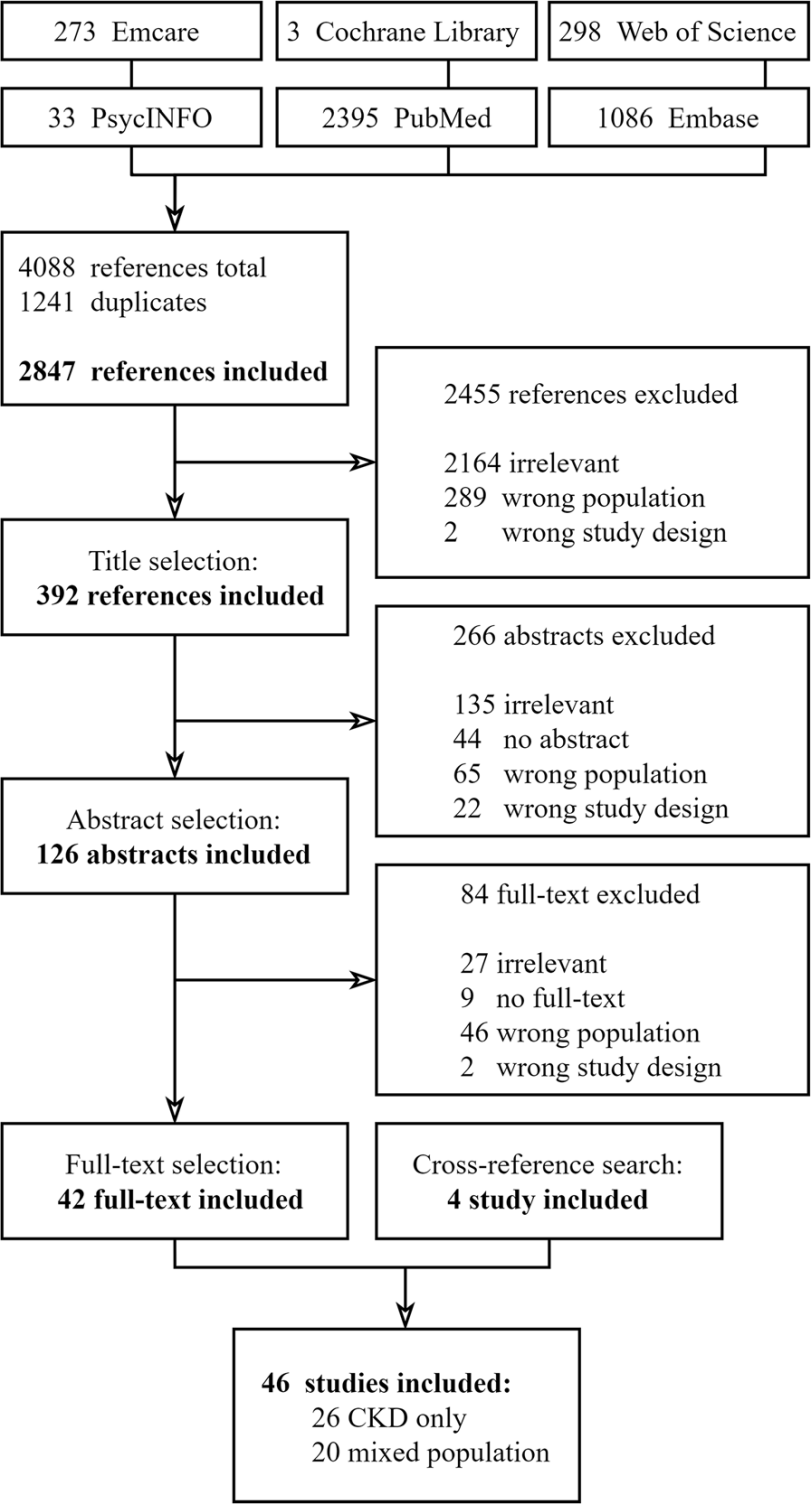
Flowchart of study inclusion. Non-qualitative studies were excluded. Studies that did not contain patients with CKD, or were mixed with other participants and of which the data were not linkable to patients with CKD were marked as ‘wrong population’. Studies that did not contain extractable data (e.g. systematic reviews), but were qualitative and included patients with CKD were marked as ‘wrong study design’. The inclusion and labelling method is described in more detail in Supplement Item 1

Of the 2847 articles identified in the search, 46 studies met the inclusion criteria, representing 1493 participants (Fig. 1). Of these 46 articles, 26 (56%) articles included patients with CKD only, including 529 participants in all CKD stages; the other 20 articles included, amongst others, patients on KRT, conservative therapy, caregivers and healthcare professionals. An overview of the 26 studies on patients with CKD is given in Table 1; the remaining 20 studies with mixed populations are presented in supplementary Table S1. Overall, studies from 12 different countries were included. Although all stages of CKD were included, most studies included participants in CKD stages 3–5.

**Table 1:** Overview of included studies. Abbreviations: SD: standard deviation, CKD: Chronic Kidney Disease; KRT: kidney replacement therapy. ^a^Studies with unspecified distribution CKD groups (e.g. “CKD 4-5 are marked as CKD 4 and CKD 5); +studies with eGFR ranges (e.g. “eGFR < 30″ are marked as CKD 4 and CKD 5) are attributed to a CKD stage according to the KDIGO CKD staging system; ^b^ mean; ^c^ median. An overview of the 20 studies that included a mixed population is presented in the supplement Table S1

	DEMOGRAPHY				CKD STAGE							RESEARCH METHODS			RESULTS
Study	Country	n.	Age	Sex m/f	1	2	3	4	5	Predialysis	Unclear	Sampling	Data gathering	Analysis	Principal research aim
Andrew, J. 2001 [36]	United Kingdom	10	–	–						x		Purposive	Interview (semi-structured)	Grounded theory	Needs and experiences of predialysis patients
Iles-Smith, H. 2005 [37]	United Kingdom	10	62^b^ (range 37–73)	8/2						x		Consecutive	Interview (semi-structured)	–	Perceptions, expectations and experiences of predialysis patients
Tweed, A. E. 2005 [35]	United Kingdom	9	54^b^ (range 29–69)	5/4						x		Convenience	Interview (semi-structured)	Phenomenology	Process of patient decision-making, perspectives and impact on life in predialysis patients
Costantini, L. 2008 [9]^a^	Canada	14	41.3^b^ (range 16–69)	6/8	x	x	x					Purposive	Interview (semi-structured)	Content analysis	Self-management, experiences and perceptions on health and support in CKD
Sakraida, T. J. 2009 [38]	USA	6	58^b^ (SD: 9.98)	4/2			x	x				Purposive	Focus group	Ethnography	Perceived resources for and barriers of self-management
Noble, H. 2010 [39]	United Kingdom	30	–	–					x			Consecutive	Observations, Interview	–	Symptoms in late stage CKD
de Brito-Ashurst, I. 2011 [40]	United Kingdom	20	60^b^ (SD: 8)	0/20							x	Purposive	Focus group, Interview (structured)	–	Views on CKD diets and salt intake and barriers and facilitators of dietary change
Nygardh, A. 2012 [41]	Sweden	20	69^c^ (range 38–86)	14/6						x		Purposive	Interview (unstructured)	Content analysis	How to empower patients with CKD
Sakraida, T. J. 2012 [42]	USA	6	58^b^ (SD: 9.98)	4/2			x					Convenience	Focus group	Ethnography	Coping resources and barriers of self-management in CKD
Walker, R. 2012 [43]+	United Kingdom	9	75.9^b^ (range 63–93)	4/5				x	x			Purposive	Interview (semi-structured)	–	Experiences with adherence to behavioural changes in late stage CKD
Johnston, S. 2012 [44]	United Kingdom	9	86^b^ (range 74–96)	4/5					x			Convenience	Interview (semi-structured)	Grounded theory	Motivation to opt for conservative therapy in late-stage CKD
McKillop, G. 2013 [45]	United Kingdom	10	60^b^ (range 29–82)	5/5							x	Purposive	Interview (semi-structured)	Thematic analysis	Views and motivations regarding adherence to medication
Lin, C. C. 2013 [11]^a^	Taiwan	15	- (range 25–77)	12/3	x	x	x					Purposive	Interview (semi-structured)	Content analysis	Illness representations and coping processes in early CKD
Lopez-Vargas, P. A. 2014 [8]	Australia	38	54^b^ (range 20–79)	23/15	x	x	x	x	x			Purposive	Focus group	Grounded theory	Experiences, perspectives and information needs in managing and living with CKD
Tangkiatkumjai, M. 2014 [34]^a^	Thailand	16	62.5^b^ (SD: 12.3)	6/10			x	x	x			–	Interview (structured)	Thematic analysis	Views on and reasons to use herbal and dietary supplements
Clarke, A. L. 2015 [46]	United Kingdom	30	FG: 68.6 (range 48–83); IV 64.1 (range 26–78)	FG: 7/6; IV: 11/6			x	x	x			Convenience, purposive	Focus group, Interview (semi-structured)	Constructivist paradigm	Motivators, barriers and beliefs regarding physical exercise
Erlang, A. S. 2015 [47]	Denmark	9	- (range 37–86)	7/2						x		Convenience	Interview (semi-structured)	Phenomenology and hermeneutics	Perspectives, values and experiences related to involvement in the choice of dialysis modality
Shirazian, S. 2016 [48]^a^	USA	23	64^b^ (SD: -)	14/9		x	x	x	x			Purposive	Focus group	Thematic analysis	Views, barriers and supports to the self-management of CKD
Wright Nunes, J. 2016 [49]^a^	USA	49	62^b^ (SD: 14)	24/25	x	x	x	x	x			Purposive	Interview (semi-structured)	Grounded theory	Emotions after diagnosis, views on how diagnosis was communicated
Wu, C. C. 2016 [50]^a^	Taiwan	15	52^b^ (range 24–81)	7/8				x	x			Purposive	Interview (semi-structured)	Content analysis	Experiences and perceptions related to living with late-stage CKD
Schipper, K. 2016 [51]^a^	The Netherlands	41	- (range 18–75)	17/24			x	x				Purposive	Interview (semi-structured), Focus group	Content analysis	Experience, needs and coping with CKD
Bowling, C. B. 2017 [12]+	USA	30	75.1^b^ (range 70.1–90.7)	29/1			x	x	x			Convenience	Focus group	Grounded theory	Self-management and complexity of CKD
Havas, K. 2017 [52]^a^	Australia	63	56.9^b^ (range 25–84)	26/37	x	x	x	x				Convenience	Interview (semi-structured)	Content analysis	Experiences, perceptions and suggestions on self-management support
Lovell, S. 2017 [33]	New Zealand	17	75.1^b^ (range 66–90)	14/3					x			Purposive	Interview (semi-structured)	Content analysis	Perspectives of progression of CKD and decision making regarding dialysis.
Pugh-Clarke, K. 2017 [53]^a^	United Kingdom	18	65^b^ (SD: 13.21)	9/9				x	x			Convenience	Interview (semi-structured)	Thematic analysis	Symptom experience in CKD stage 4 and 5
Campbell-Crofts, S. 2018 [10]^a^	Australia	12	- (range 31–81)	4/8			x	x	x			Convenience	Interview (semi-structured)	Thematic analysis	Views on decision making regarding KRT in late stage CKD

### Synthesis

**Fig. 2:**
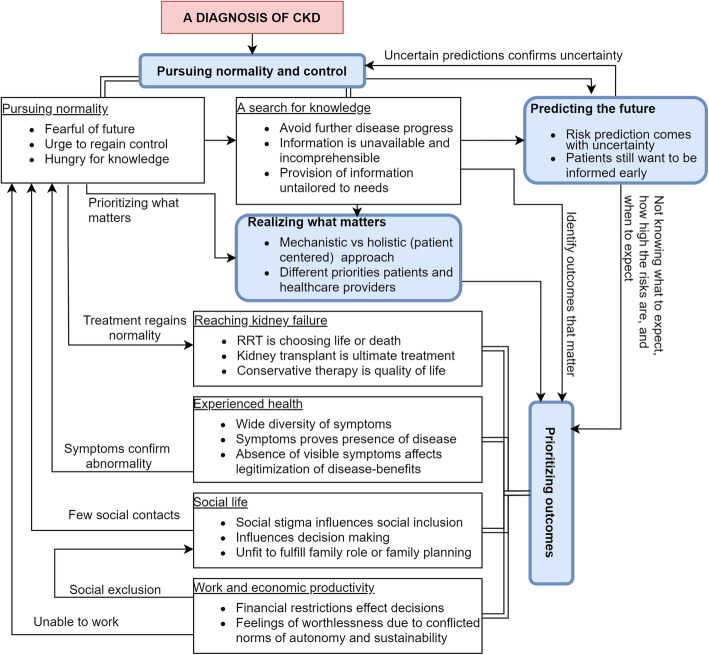
Thematic schema: an overview of the identified themes with a hypothesized relation between themes. Patients with CKD face uncertainties and problems regarding their disease progress. This is aggravated by the lack of knowledge, incomprehensible and unavailable information and impossibility to adequately estimate risks, essentially leaving patients in a situation where they do not know what to expect, how high the risks are, and when to expect certain outcomes of interest. Major themes (blue boxes, bold) are linked to subthemes (white boxes, underlined). Abbreviations: CKD; chronic kidney disease, KRT; kidney replacement therapy

In total, 4 main themes and 6 subthemes were identified (see Table 2 and Fig. 2).

**Table 2:** Overview of the major themes and subthemes with illustrative quotations

Theme	Illustrative quotations
Pursuing normality and control	1) Subtheme: pursuing normality ● *“It was with the nurse and she said ‘what do you want out of life?’ And I said ‘I still want to be able to drive and I still want to be able to play golf if possible’. And looking at the [information] booklet she gave me, that [CAPD] looked about the only thing I could do but it’s not going to mess my life about any more than I have to. Really trying to keep it at bay. It’s there but push it in the corner.”* [35] ● *“Yeah, I’m considering peritoneal dialysis because it interferes with your life less. You can do it at night. And it doesn’t interfere with your day... If you do it overnight, all your days are free.”* [54] ● *“I don’t know what it’s like to be normal anymore, to feel normal.”* [55] 2) Subtheme: a search for knowledge: ● *“The more information I have, the more knowledge I got. That means I can ask better questions, more intelligent questions … otherwise I didn’t have a chance to process it. ”*[38] ● *“(…) [I] shouldn’t have to try and read all this medical jargon cause I’m not a—I’m an artist. I’m a painter. I don’t know what this means and that means.”* [48] ● *“We didn’t take 4 years of Latin. An even if we did, it’s so far back that we don’t remember anymore, and we didn’t have medical. So you got to bring down to the level of understanding for the normal person. If it’s a kidney, call it a kidney. ”*[38]
Prioritizing outcomes	1) Subtheme: reaching kidney failure ● *“When they say I’ve got to go on [dialysis] then I’ll work it in, because I’ve got no choice. It’s either that or die.” [laughs]* [33] ● *“It was like a monster kind of waiting and lurking in the dark for me and I didn’t like the idea at all. Being dependent on the machine for all the functions that you were doing naturally since you were born and the machine takes over and there’s no way back. You are not free anymore to make any decisions. If you want to go away it takes so much planning. You are strapped to a machine. ”*[56] ● *“I’m afraid of receiving dialysis… I want to use everything, which helps me to avoid receiving dialysis.”* [34] 2) Subtheme: experienced health ● *“If I’m going to feel this bad for the rest of my life, do I just want to end it now?”* [57] ● *“It’s a strange kind of tiredness, quite unlike anything that I’ve had before. You can’t really describe it … it’s weird. You just sit down and, phew, you’re gone [fallen asleep]. It’s weird, strange. ”*[53] ● *“My thought processes seem to be slowing ”*[53] 3) Subtheme: social life ● *“Cultural too, is the male working thing, the identity of working and being a working man, and the stigma of being sick and on dialysis and not being the tough guy ”*[58] ● *“The nephrologist is more about making sure the kidney doesn’t fail or making sure I live as long as possible, whereas I’m willing to accept some risks for happiness—having a family. ”*[59] ● *“I can be afraid if I think about the future … Will he still love me if I have more restrictions? And can we stay partners on equal terms? “*[51] 4) Subtheme: work and economic productivity ● *“There’s no way I can go back to working where I used to, there’s no way I can stand on my feet for 8 h doing the heavy work I used to do, there’s all the retraining and going back into the workforce, plus trying to work out how I’m going to pay my bills, my rent. ”*[55] ● *“Doing a lot of things that I was able to do six years ago, I can’t do that and that’s really frustrating, you know, for me because, as my kids know, I worked all my life. I managed a restaurant for 37 years and supported 7 kids … and now I can’t work. It’s frustrating that I want to go out there and work, do something to help keep me going, and my kids, and I know I can’t … Mentally it’s like ‘Why am I here if I can’t do anything to help?’ “*[42] ● *“My colleagues and employer don’t know that I have CKD. I’m afraid they will use it against me”* [51].
Predicting the future	● *“He said to me ‘Look, you’ve got a GFR … falling, it is at 22 now which means that you’ve got about a year left before it’s dialysis or transplant.’“* [10] ● *“At the moment he’s sitting on the, on his, hands and saying ‘Well, it doesn’t look like it [dialysis]‘ll be happening until sometime next year.’“* [33] ● *“The notion that it will be more difficult in the future is always there. I may not have many problems right now, but the sword of Damocles is always hanging over my head. ”*[51]
Realising what matters	● *“For the last year and half, I’ve been asking my doctor to change my medications so we can have a child and they keep saying ‘next appointment, next appointment.’”* [59] ● *“There is really nothing to discuss with the doctor. [...] the doctor is wary and persuaded me to accept dialysis [...] all they would do is to encourage me to go on dialysis and tell me the benefits of dialysis.”* [60] ● *“He [name of nephrologist] brought up dialysis and was asking me whether I want to have peritoneal dialysis or haemodialysis. During that conversation we seemed to conflict with each other, so what I thought was one thing, he said, ‘No, no, no, that’s not what you want…’ and I’m like ‘No, I’m pretty sure I want that’.”* [10]

## Pursuing normality and control

This theme comprises two subthemes: *pursuing normality* and *a search for knowledge*; both describing the need for certainty.

### Pursuing normality

The gravity of being diagnosed with a life threatening disease, and the realization that with progression of time and decreasing kidney function various outcomes may occur, was unsettling for most: *“Having CKD is just like walking in the valley of the shadow of death, and I can see no hope … My children are still so young. Death has cast a shadow over me, and I am very affected.”* [11]. In this disruptive and bewildering period, patients reached a moment where they felt the need to regain control of their disease and return to normality [8, 17, 36, 48, 50, 52, 54, 55, 60–63]: *“If you can’t have some semblance of a normal life, then why would you want to live”* [55]. Especially in the earlier phases of CKD, when few symptoms were experienced, feeling normal instead of feeling like a patient with a chronic disease was relatively easy. However, with the increase of disease severity, participants became more aware of their disease, and expressed an urgency to regain control and stop further deterioration of their health. Patients employed various self-regulation and coping strategies, with searching for information as the main recurring strategy.

### A search for knowledge

Patients try to gain insight into and understand their disease in order to get a “grip on it”. Many patients expressed a great interest in the mechanisms of the disease [8, 10, 37, 41, 43, 47, 51, 58, 64–67], and methods to avoid further kidney function deterioration. However, this search for information turned out to be a frustrating experience, as patients felt readily accessible and understandable CKD-related information was lacking [38, 41, 53, 66, 68, 69]. *“I think what is missing from most of these [brochures] is – WHY? They tell you about it but they don’t give you the reason why it’s like this.”* [8] Appointments with healthcare professionals were often regarded as stressed and hurried [8, 9, 12, 38, 40, 47, 49, 51, 58, 70], and the information received as conflicting [8, 12, 48, 51, 58, 65], insufficient [9, 50, 59, 65], unclear [38, 47, 49, 61, 68, 69, 71, 72], too much [42, 58, 71], too unspecific and untailored to their situation [9, 11, 12, 40, 45, 53, 62, 65, 72], or too late [9, 44, 53]. Subsequently, patients turned to other sources for information, including peers [11, 42, 45, 48, 55, 61, 62, 64, 67], family members [11, 55, 59, 60, 72], friends [55, 59–61, 63, 72], online health information [8, 9, 11, 49, 66] or mass media [11, 63]. Consequently, knowledge on the CKD trajectory towards kidney failure was largely anecdotal, incomplete and not well understood [11, 38, 63, 64, 67, 72]: *“I have seen my friends go through dialysis and the shows on television. The people on dialysis look so weak and helpless.”* [63]. Especially in the absence of symptoms, patients felt no urgency to pursue knowledge on CKD facing these difficulties. However, patients that were content with information provided by healthcare professionals felt generally more empowered, confident, in control, and felt responsible for their own health [38, 40, 44, 47, 49, 51, 58, 65, 72]: *“I think it’s interesting to know as much as I can about my illness. I mean, the more you know about it, the more chance you’ve got to influence how it goes – and you’re prepared in quite another way for what might happen. That’s more or less how I see it.”* [47].

## Prioritizing outcomes

Outcomes prioritized by patients could be grouped into four subthemes (*reaching kidney failure, experienced health, social life*, and *work and economic productivity*), which describe outcomes both directly related to the disease and more personal outcomes.

### Reaching kidney failure

Although patients prioritized many different outcomes, reaching the moment when KRT initiation would be necessary was predominantly felt as important by most patients [8, 10, 11, 36, 38–44, 48, 51, 53, 54, 59–62, 64, 67, 68, 73, 74]: “*(…) I may not have many problems right now, but the sword of Damocles is always hanging over my head”* [53]*.* It was described as a disastrous, inevitable and constantly looming possibility [8–11, 17, 37, 44, 53–55, 59–61, 64, 66, 68, 74, 75]. Choosing for KRT was often expressed as choosing between life and death [11, 37, 54, 73, 75], with receiving a kidney transplant seen as the ultimate treatment [39, 40, 43, 48, 52, 64, 76] or, as a patient phrased it: “*(…)the only chance to have a normal life*” [64]. In contrast, dialysis was often regarded as the opposite of quality of life [8, 10, 38, 41, 52, 54, 55, 61, 63, 67, 73, 75] or an early sign of dying [41, 52, 59, 63, 75] while conservative therapy was regarded as choosing for quality of life instead of pointless prolonging [8, 54, 61, 63, 75].

### Experienced health

Patients experienced a range of symptoms that were either associated with CKD itself, the disease that caused CKD (e.g. diabetes), treatment side effects, or other comorbidities. In 29 out of 46 articles, a total of 77 different symptoms were mentioned, with the number of symptoms per article ranging between 1 and 50 (presented in supplementary Table S3). Fatigue and a general feeling of weakness was mentioned by many patients in most articles, although it proved to be difficult to describe to others: *“(…) a feeling, not something obvious. With chronic kidney disease, you don’t look different. They tell you, you look good, but they don’t see what’s inside.”* [48]*.* Fatigue was also often regarded as something normal and consequently, patients felt estrangement and an urge to convince others about the disease severity [48, 53, 62, 65].

### Social life

Living with CKD affected patients’ social circles, including family and friends. Some effects were practical in nature, such as burdening family with logistics of CKD and treatment, dialysis preparations or being unable to perform daily tasks [8, 10, 17, 41, 43, 48, 53–55, 63, 64, 73, 75, 77]. In some cases, these practical concerns influenced decision making, e.g. regarding starting with dialysis [42, 63, 75]. Living with the consequences of a chronic disease and associated physical and medical restrictions, affected social inclusion and patients’ ability to partake in certain social occasions, such as dinner with family or friends [9, 12, 38, 41, 43, 48, 51–54, 58, 66, 70, 72]. *“You know, my wife says to me now, you know, we’ve lost a lot of friends because of my condition, because I’ve been moody or I get moody, you know. People don’t understand what you feel or what you’re going through.”* [58]. Being unable to fulfil the same role within the family as before CKD was diagnosed caused considerable anxiety amongst patients [11, 48, 53, 59, 67]: “*You don’t live the life you would like to live. I can’t lead the life I envisioned for myself and my kids. (…) I’m just trying to survive*.” [53] Some patients also mentioned the effects of their disease on their sexual wellbeing and family planning [38, 47, 48, 51, 53, 59, 70, 73, 76]:*“You are not a real man anymore because of your decreased libido. It feels as if I have failed”* [53]. While patients experienced changes in their social role, they also noticed that others changed their behaviour towards them. Although patients expressed the desire that their social circles took their symptoms serious, they lamented the social stigma surrounding CKD and felt like they were often solely being regarded as a patient instead of the person they once were [8, 48, 53, 64, 67, 73]: “*I don’t want to have the “stamp” patient, because I don’t feel like a patient right now”* [53]*.*

### Work and economic productivity

Being unable to sustain a full-time job resulted in feelings of uselessness. This was emphasised in the absence of clear visible symptoms, and thus legitimisation of disease, by their employers or colleagues, as it conflicted with perceived norms of autonomy and sustainability [8, 11, 39, 48, 53, 59, 73]. The effects of CKD on financial independence was often mentioned by patients [8, 39, 48, 53, 58, 59, 63, 73] and influenced decision making in some cases [45, 48, 58, 64]: *“With home dialysis, I can work more and support my family and that’s really important cause they are reliant on me financially”* [45]*.* Also, being unable to work was reported to decrease social involvement and acceptance [48, 53]: “*Conversations at parties stagnate when you say that you don’t work*.” [53].

## Predicting the future

Patients were interested in both the risk of reaching the outcomes that matter to them, but also the timeframe until these outcomes might occur –indicating that both estimates are important for care planning. Although in some studies patients were given an indication of risk by their healthcare professional [8, 10, 36, 40, 51, 53, 54, 68, 74], they understood the uncertainty: *“I’ve got a rough timeframe, again its imperfect so no one knows definitively. People say ‘When are you going on dialysis?’ Well no-one knows but we can guess the way it’s going, we can guess”* [10]. This left patients in a position where they did not know which outcomes to expect, how high risks for these outcomes were, and when this outcome might occur. This uncertainty regarding their future was accompanied with anxiety, frustration or even resignation to regain control on their disease [8, 10, 38, 40, 48, 64, 65, 67, 76]. Yet, despite the uncertainty about the future, patients expressed the need to be informed as early as possible on their trajectory nonetheless [9, 10, 37, 44, 49, 50, 53, 55, 67]: *“The nephrologist advised me not to think about dialysis or transplantation yet because I’m not in that stage of the disease yet. But I know I will need it one day so it’s not that easy to put all those emotions and doubts away”* [53]*.*

## Realising what matters

On top of living in a vacuum of prognostic uncertainty, many patients described being misunderstood by their healthcare professionals. Although the exact instances varied widely, there were two main reasons patients felt unheard: 1) professionals displayed a mechanistic approach to disease without an interest in forming relationships, instead of a holistic and person centred approach [9, 10, 17, 40, 41, 43, 47–49, 53, 65, 66, 68, 75, 76]: *“I want to be more than my renal function. They don’t see you as a person*.*”* [53], and 2) a difference in priorities between healthcare professionals and patients [8–10, 12, 37, 40, 53, 54, 63, 67, 68, 76, 78]: *“My nephrologist just saw kind of being pregnant as an associated risk, not really as a human thing.”* [76]. The feeling that not they, but the healthcare professionals were in control of their disease trajectory often resulted in frustration and alienation. Nevertheless, the ‘*ultimate decision*’ [48] whether or not to start KRT was often left in the hands of, or at least influenced heavily by, their healthcare professionals [10, 11, 40, 42, 47, 51, 53, 54, 61, 66, 68, 76]: *“I am an independent person and I would like to decide about most things. But I also think that if somebody comes and says this is a really bad decision you have made because this, and that and this is supported by arguments then well, I give in to that.”* [40].

### Comprehensiveness of reporting

**Fig. 3:**
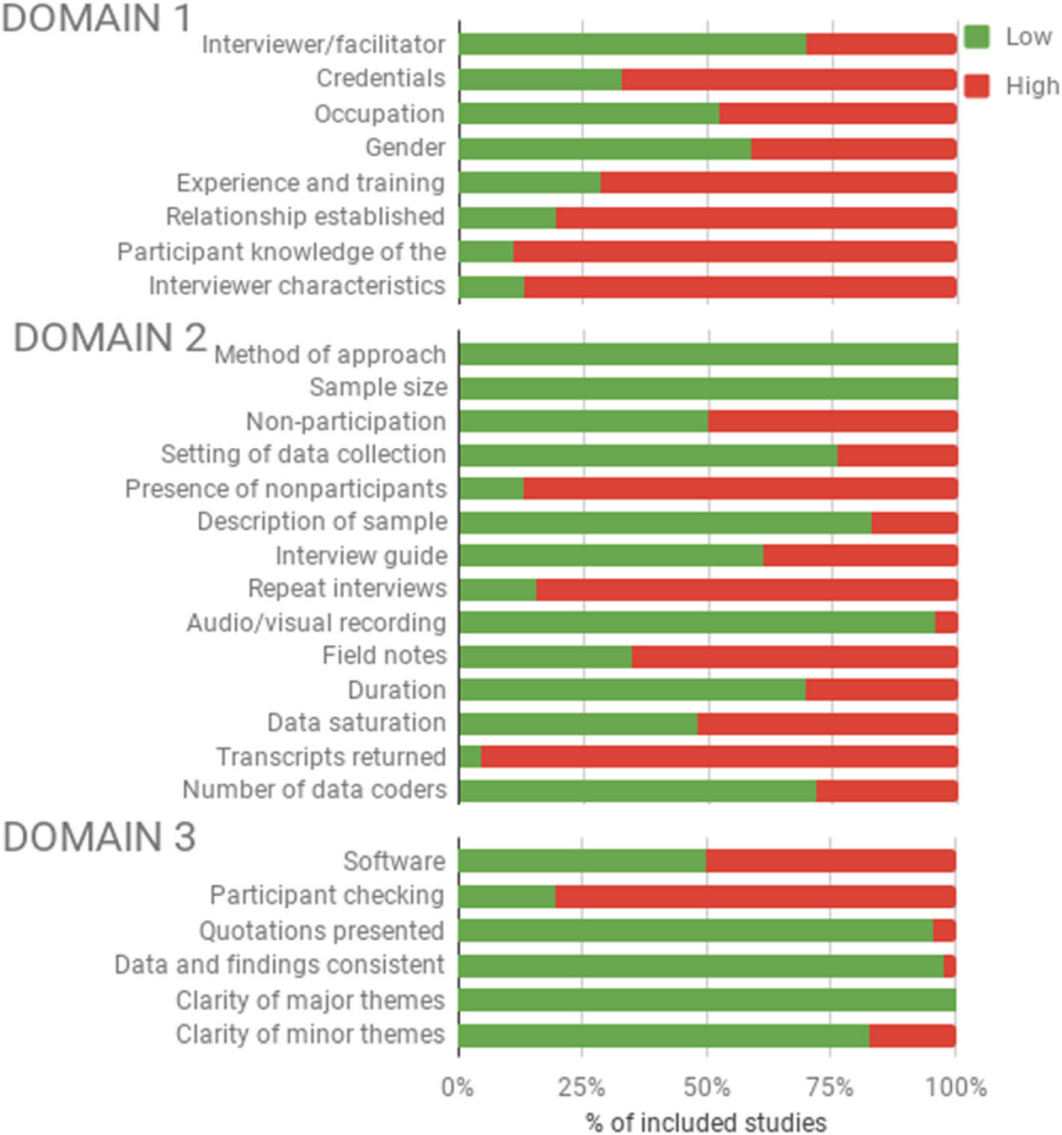
COREQ quality of reporting summary of the 46 included studies, over the three domains (domain 1: ‘research team and reflexivity’, comprises 8 signalling questions which describes both the personal characteristics of the researchers and their relationships with the participants; domain 2: ‘study design’ comprises 15 questions which describes the included population and study methods; and domain 3: ‘analysis and findings’, comprises 9 questions which describes the analysis and clarity of the results) containing a total of 32 signalling questions. An overview of each individual study is presented in the supplementary Table S2

The completeness of reporting as assessed by the COREQ was moderate, with studies reporting between 8 to 25 of the 32 items, averaging 18.6 items (58%). A total of four studies scored very good (≥25 items), 30 scored moderate (17–24 items), 11 scored poor (9–16 items) and one scored very poor (≤8 items). Reporting quality was especially weak with regard to describing the domain ‘research team and reflexivity’ (average 2.8 out of 8), followed by the domain ‘study design’ (average 9.2 out of 15) and finally, the domain ‘analysis and findings’ (average 6.5 out of max. 9). A summary of the quality of reporting per domain is shown in Fig. 3. A detailed overview of each study is presented in supplementary Table S2.

## Discussion

In this thematic synthesis of 46 qualitative studies, we explored the priorities regarding outcomes of patients with CKD and barriers encountered regarding person-centred care and care planning. The themes that emerged describe the health journey after diagnosis with CKD, underline the disruptiveness of CKD on all aspects of life, and the urgency felt for incorporation of person-centred care within routine medical care. We identified four major themes with six subthemes: *pursuing normality and control* (subthemes: *pursuing normality*; *a search for knowledge*); *prioritizing outcomes* (subthemes: *reaching kidney failure; experienced health; social life; work and economic productivity*); *predicting the future* and *realising what matters*. Three barriers relevant to person-centred care provision were embedded within these themes: untailored and incomprehensible information, the inability to accurately estimate risks, and differences in priorities regarding outcomes and care processes between patients and healthcare professionals. The overall completeness of reporting as assessed by the COREQ was moderate, especially so for the domain ‘research team and reflexivity’.

Following the disrupting period after diagnosis of CKD, patients express the need to return to normality and regain control to avoid further deterioration of kidney function and associated physical and mental symptoms. However, as patients in early stages of CKD usually experience few symptoms, the initial shock of being diagnosed with a chronic disease subdues, and maintaining or ignoring the status quo turned out to be relatively easy. A complicating factor in regaining control was clearly described by many patients in our study, namely the struggle and frustration to gain comprehensible information tailored to their specific situation, which we identified as an important barrier for person centred care provision. As a consequence of both the absence of symptoms and the difficulties in obtaining relevant information, self-management strategies were postponed by patients. The delayed self-management activation but also the strive for normality in the earlier stages of disease are not unique to CKD, but are observed in other chronic diseases as well [79, 80].

As time passed and disease progresses, patients with CKD learn about, or in some instances already experience some of the possible outcomes related to CKD - both directly related to the disease, such as physical or mental symptoms, but also indirectly such as a social stigma or financial burdens. Prioritization of these outcomes differed greatly between the patients in our study, but one outcome was emphasized and feared most: reaching kidney failure and choosing between dialysis, transplant or conservative care. Although discussing this topic is regarded as a difficult subject, both by clinicians and patients, it is often recognized as an important subject and thus prioritized and facilitated by healthcare professionals. This is in contrast to the other three groups of outcomes which were regarded by some patients as equally important: apart from kidney failure, patients worry about the symptoms associated with CKD – both physical and mental symptoms, the effects of CKD on their social life and on economic productivity. These other aspects of disease are not routinely assessed by healthcare professionals and often go unnoticed as a result. Consequently, patients feel misunderstood by their healthcare professionals, as they realize that their priorities do not match those of their healthcare professionals. Indeed, many patients in our study expressed the need for holistic care, instead of an approach they described as mechanistic: a focus on laboratory results instead of their actual perceived wellbeing. This barrier for person centred care was mentioned in most studies, and caused considerable frustration with, and alienation from healthcare professionals. Although healthcare professionals are aware of the disruptive effects of CKD on these important aspects of life [18, 81], traditionally the main focus of care is on prolongation of time to kidney failure or death [81]. Illustrative, in a 2003 survey, US nephrology fellows reported that palliative care training was not integrated sufficiently in their curricula, and consequently they felt unprepared to discuss end-of-life issues [82]. Despite that, and even though the majority of our included articles have been published in between, a repeat survey 10 years later showed similar results [83].

Patients have a realistic expectation that neither the risks of future outcomes nor the timeframe of reaching them can be predicted with a large degree of certainty. This realization causes feelings of anxiety and frustration, and consequently, we identified this as the third and final barrier for person centred care implementation. Yet, despite the uncertainty of the risk estimates, participants in our study expressed eagerness to be informed as early as possible, and urged not to withhold information on prognosis. We identified many instances where selective, delayed or incomplete information on sensitive topics such as disease progression or kidney failure frequently resulted in frustration and in some cases even mistrust. Clinicians are aware of this prognostic uncertainty, but refrain from discussing risks because they lack aids to adequately counsel patients on the outcomes of their interest [84], or fear to emotionally overwhelm patients [81, 85]. However, deciding early and planning in advance which treatment option is most suitable or which outcomes to avoid, has been shown to positively enhance patients’ coping with disease [86], especially when the preferences of patients are taken into account [87]. This relation between risk uncertainty and focus on kidney failure or prolonging survival is illustrated for example by the number of prognostic prediction models that have been developed for these outcomes in patients with CKD: for KRT and death respectively 42 and 16 models were identified in systematic reviews [88, 89], and models validated in these populations perform poorly [90–92]. In contrast, no models for other outcomes prioritized by patients exist. Interestingly, contrasting the number of prediction models on this topic, the risk of death was mentioned only a few times by patients, usually in the context as an alternative for KRT, i.e. conservative treatment [18].

Our study provides several clinical implications. Patients were frustrated about the lack of available and accessible information, and realize that disease education is not prioritized by their healthcare professionals. Consequently, they look for information elsewhere, resulting in incomplete or even incorrect information. Several systematic reviews on patient education and self-management have demonstrated positive effects of education on knowledge and self-management, though the number of included studies was low and the effects dependent on the type of educational interventions and setting [79, 93, 94]. Studies in other chronic diseases, such as diabetes [95, 96] and hypertension [94, 97] demonstrated similar results. Our findings thus underline the importance of disease education in CKD. Next, patients with CKD describe the wide array of problems they experience, but feel unheard and misunderstood by their healthcare professionals. For example, one of the recurring themes was the influence of CKD on work and economic productivity. Conversations between healthcare professionals and patients might stimulate that healthcare professionals support patients in coping with work related concerns, make appropriate referrals to a social worker, or help patients arranging a more flexible work environment. Another finding was the struggle with a wide array of disease related physical and mental symptoms of CKD - we identified a total of 77 distinct symptoms -, which often remained unnoticed by their healthcare professionals. Our findings could thus serve as a guide for identification of care needs for healthcare professionals. More formally, our study could be used as a starting point in the development or selection of PROMs and incorporation of these PROMs within routine CKD care [57]. Incorporation of person-centred care and PROMs in CKD shows promising results [23–25] and may result in outcomes that are more satisfactory [98]. Finally, patients realize that the prediction of outcomes of interest is largely impossible. Prediction studies on the development of kidney failure or the risk of death have been conducted, however these cover only a small part of the spectrum of outcomes that are important to patients. Future prediction studies could focus on other patient-prioritised outcomes (for examples predicting outcomes such as ‘when will I have to give up work?’ or ‘when will I be unable to drive?’) or on predicting PROs: similar studies have been conducted in orthopaedics [99–102], neurosurgery [103], and psychiatry [104].

Our study comes with strengths and weaknesses. This is the first study to comprehensively provide an overview of outcomes prioritized by patients with CKD and barriers for person centred care provision by means of a systematic review of qualitative studies, using a broad scope by not focussing on the medical side alone. Thematic synthesis of qualitative studies instead of original data is a relatively novel method to achieve abstraction and transferability at a higher level beyond the included original studies [22]. Another strength of this study is the inclusion of a large number of patients in all stages of CKD, from many different countries including a diverse demographic and many different ethnicities. Our study is however not without its limitations. First, without inclusion of non-English articles, transferability to non-English speaking populations is unclear, although we included several articles with quotes that were translated to English. As indicated with the COREQ criteria, most of the included studies incompletely reported information on their methodology or findings, which may have impacted the validity of our results. As this is not uncommon in qualitative research [105], and because the aim of our study was to ensure a broad range of perspectives were captured and to encourage transparency and transferability of findings, we included all studies regardless. Next, because most studies were conducted in developed countries, the transferability of our findings to developing countries is uncertain. Finally, as with all qualitative research, interpretation and reporting of findings is influenced by the personal beliefs and biases of the researcher (i.e. research reflexivity). To prevent that data interpretation and results were strongly coloured by the preconceptions of a single profession, we purposely created a team of authors with a wide diversity of professional background and experience.

## Conclusion

Living with a diagnosis of CKD has a major impact not only on physical outcomes, but on many other aspects as well, such as mental health, social life and emotional wellbeing. Inadequate provision of information tailored to both the stage of the disease and the capacities of the patient, uncertainty regarding the prognosis and difference in priorities between healthcare professionals and patients are barriers that stand in the way to optimal person-centred healthcare. Multidisciplinary care and regular use of PROMs in nephrological care may be a strategy to help focus care on the needs and outcomes of most importance to adults with CKD.

## Supplementary Information

**Additional file 1: Fig. S1** labelling strategy for study inclusion, showing the four consecutive steps of study inclusion. **Table S1.** Overview of the 20 studies with mixed populations. **Table S2.** Quality of reporting of the 46 included studies, per individual study, as assessed with the COREQ. If a topic is mentioned in the individual study, this is marked green; if not, it is marked red. **Table S3.** Overview of symptoms mentioned in the included 46 articles. Using a deductive approach, symptoms were grouped into categories. In some cases, a more general term is used to describe certain symptoms for brevity, e.g. ‘appetite’ was used to describe increased or decreased appetite; ‘skin’ was used for dry, weak or brittle skin, etc.

## Data Availability

The datasets generated and/or analysed during the current study are not publicly available as it consist of publications (for this systematic review), each with different copyrights, but are available from the corresponding author on reasonable request.
