# Supplementary material for: Person centred care provision and care planning in chronic kidney disease: which outcomes matter? A systematic review and thematic synthesis of qualitative studies: Care planning in CKD: which outcomes matter?
Source: BMC Nephrol. 2021 Sep 13;22:309. doi: 10.1186/s12882-021-02489-6 (PMC8438879; doi:10.1186/s12882-021-02489-6)
Supplement: Supplementary file 1 — Additional file 1: Fig. S1 labelling strategy for study inclusion, showing the four consecutive steps of study inclusion. Table S1. Overview of the 20 studies with mixed populations. Table S2. Quality of reporting of the 46 included studies, per individual study, as assessed with the COREQ. If a topic is mentioned in the individual study, this is marked green; if not, it is marked red. Table S3. Overview of symptoms mentioned in the included 46 articles. Using a deductive approach, symptoms were grouped into categories. In some cases, a more general term is used to describe certain symptoms for brevity, e.g. ‘appetite’ was used to describe increased or decreased appetite; ‘skin’ was used for dry, weak or brittle skin, etc. [file 12882_2021_2489_MOESM1_ESM.docx]

SUPPLEMENTARY MATERIAL FOR

**Person centred care provision and care planning in Chronic Kidney Disease: which outcomes matter? A systematic review and thematic synthesis of qualitative studies.**

Subtitle: Care planning in CKD: which outcomes matter?

Ype de Jong1,2, Esmee M van der Willik1, Jet Milders1, Yvette Meuleman1, Rachael L Morton3, Friedo W Dekker1, Merel van Diepen1

1. Department of Clinical Epidemiology, Leiden University Medical Centre, The Netherlands¬¬

2. Department of Internal Medicine, Leiden University Medical Centre, The Netherlands

3. NHMRC Clinical Trials Centre, Faculty of Medicine and Health, The University of Sydney, Australia

Correspondence to:

Ype de Jong, department of Epidemiology, Leiden University Medical Centre, Albinusdreef 2, 2333 ZA Leiden, The Netherlands. Y.de_jong@lumc.nl

**INDEX:**

**Search strategy and detailed risk of quality of reporting assessment**

Page 2 Detailed search methods, pilot search

Page 3 Figure S1: labelling method

Page 4 Table S1: Overview of the 20 studies with mixed populations

Page 5-6 Table S2: detailed quality of reporting assessment (COREQ)

Page 7-9 Table S3: overview of symptoms experienced by patients

**Search strings:**

Page 10 PubMed search string

Page 11 Embase search string

Page 12 Web of Science search string

Page 13 Cochrane Library search string

Page 14 PsycINFO search string

Page 15 Emcare search string

**Other:**

Page 16-20 References of included studies

**Detailed search methods, pilot search**

A systematic search was conducted on 24-04-2018 in PubMed alone, which yielded 1799 references. The eligibility criteria were:

1. Study population consisted of adults (>18y; younger patients were excluded because of different implications in shared decision making) with CKD stage 1 to 5.
2. Study methodology must be qualitative (e.g. interviews, focus groups, participants observations, case studies) and not quantitative (i.e. data should be extractable and usable for thematic synthesis)

As our main aim was primarily to identify all qualitative articles on CKD, there were no other sex, age, ethnicity, or comorbidity restrictions. Studies with mixed populations (e.g. patients with CKD, patients on dialysis, transplanted patients) were included if the qualitative data could be separated out. Discussion between the authors was held in advance to identify relevant keywords.

**Refining final search**

We piloted our search by sequentially and independently selecting the first 250 titles, followed by the abstracts and full-text for eligibility. Of these 250 references, 12 full-text were found to be eligible. Cross-reference searching of these 12 studies resulted in another 14 studies that were also cross-referenced, yielding a total of 26 studies.

Subsequently, to test the quality of our PubMed string, these studies were checked against the PubMed search. 10 of the 26 studies (38%) were not included in our search, but 3 of those 10 were not indexed in PubMed and were therefore discarded to sharpen the search, resulting in 7 of the remaining 23 studies (30%) that were not included in the search. We checked the titles and abstracts of these 7 studies and added several keywords, including “Qualitative, Perception, Coping, Barrier, Focus group”. 20 of the 23 studies (86%) were now included in the PubMed search, which we deemed appropriate. The search was then transformed to Embase (n=1086), Web of Science (n=298), Cochrane Library (n=3), PsycINFO (n=33), Emcare (n=273) and again on PubMed (n=2395). This final search was conducted on 18-07-2018 and resulted in 2847 references after removal of duplicates.

**Study selection, labelling strategy**

After excluding the previous 250 references used in the pilot search, two authors independently checked titles, abstracts and full-text on eligibility based on the inclusion criteria. Excluded articles were labelled with predefined labels:

1. 'no abstract or full-text', meaning that no abstract or full-text could be identified after consultation of a medical librarian.
2. 'irrelevant', meaning that the study was not of qualitative methodology (this did not include reviews on qualitative studies).
3. 'wrong population', meaning that the population was either not a CKD population, or a mix of CKD with other populations in which the data could not be separated out (e.g. the quotes could not be linked to a CKD patient).
4. 'wrong study design', essentially all articles that were of qualitative methodology, on CKD patients, but without extractable data (e.g. reviews, editorials, patient leaflets, etc.).

This labelling strategy is shown in figure S1.
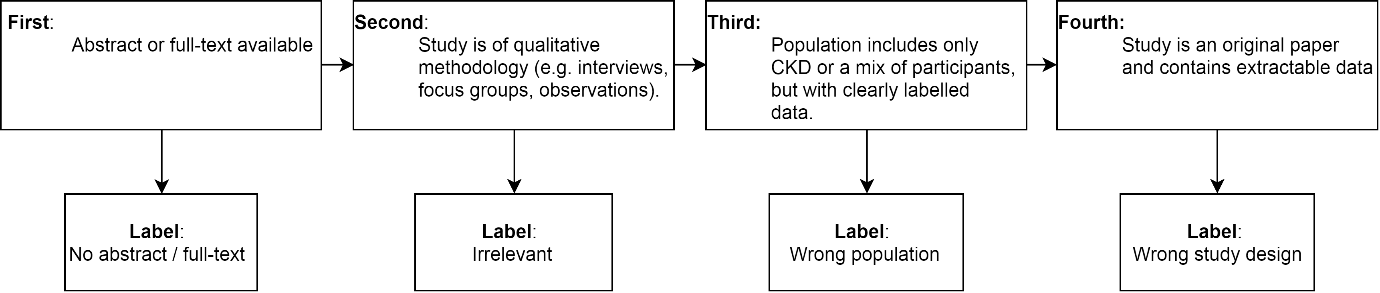


**Figure S1** labelling strategy for study inclusion, showing the four consecutive steps of study inclusion.

In the end, a total of 42 articles were included using these combined and comprehensive searches. References of all included articles, excluded but relevant reviews and editorials were checked, and subsequently checked for eligibility by abstract and full-text. Using this method, we added another 4 articles to the 42 articles that were identified with the systematic searches, yielding a total of 46 studies.

**Table S1** Overview of the 20 studies with mixed populations.

| DEMOGRAPHY | | | | | CKD PATIENTS  (stage) | | | | | | | RRT PATIENTS (type) | | | | | Others | | | | RESEARCH METHODS | | | |  |
| --- | --- | --- | --- | --- | --- | --- | --- | --- | --- | --- | --- | --- | --- | --- | --- | --- | --- | --- | --- | --- | --- | --- | --- | --- | --- |
| Study | Country | n. | Age | Sex m/f | CKD1 | CKD 2 | CKD3 | CKD4 | CKD5 | Predialysis | Unclear | HD | PD | CAPD | Unclear | RTx | Healthcare | Relatives | Children | Unknown | Sampling | Data gathering | Analysis | Principal research aim | |
| Beard, B. H.  1969^1^ | USA | 14 | -  (range 15-49) | 8/6 |  |  |  |  |  | x |  |  |  |  |  |  |  |  | x |  | Consecutive | Interview (unstructured) | Biographical | Fear of death and fear of living with CKD and the prospect of RRT | |
| Tong, A.  2008^2^ | Australia | 63 | 52.3†  (range 20-78) | 30/33 |  |  |  |  |  | x |  | x | x |  |  | x |  |  |  |  | Purposive | Focus group | Thematic analysis | Patients’ perspectives on priorities for health research in CKD | |
| Lee, A.  2008^3^ | Denmark | 45 | 54†  (range 25-87) | 15/12 |  |  |  |  |  | x |  | x | x | x |  |  |  | x |  |  | Convenience | Focus group | - | Patients’ experiences and views regarding choices on dialysis modality | |
| Tong, A.  2009^4^ | Australia | 63 | 52.3†  (range 20-78) | 31/32 | x | x | x | x | x |  |  | x | x |  |  | x |  |  |  |  | Purposive | Focus group | - | Experiences and perspectives on CKD, its treatment, its impact on lifestyle and relationships, and coping strategies | |
| Boulware, L. E.  2011^5^ | USA | 16 | NS  (range 34-71) | 7/9 |  |  |  |  |  | x |  |  |  |  |  |  |  | x |  |  | Purposive | Interview (structured) | - | Perceived barriers to discuss pre-emptive transplantation and views on the value of trained professionals to address barriers | |
| Morton, R. L.  2011^6^ | Australia | 34 | 60†  (range 30-86) | 17/17 |  |  |  |  |  | x |  | x | x | x |  |  |  | x |  |  | Purposive | Focus group | Thematic analysis | Priority characteristics of dialysis for decision making on treatment | |
| Llewellyn, H.  2014^7^ | United Kingdom | 19 | -  (range 73-94) | 12/7 |  |  |  |  | x |  |  |  |  |  |  |  |  |  |  |  | Purposive | Interview (semi-structured) | Phenomenology | Experiences of older patients on living with CKD and treatment choices | |
| Meuleman, Y.  2014^8^ | The Netherlands | 48 | 60.6†  (SD: 11.7) | 22/26 |  |  |  |  | x |  | x |  |  |  |  | x | x |  |  |  | Purposive | Focus group | Thematic analysis | Perceived barriers and support strategies for reducing sodium intake | |
| Lederer, S.  2015^9^ | USA | 32 | 63†  (SD: 9.55) | 30/2 |  |  | x | x | x |  |  |  |  |  | x | x |  |  |  |  | Purposive | Interview (semi-structured) | Thematic analysis | Barriers to addressing CKD patients’ information needs | |
| Seah, A. S.  2015^10^ | Singapore | 9 | 81‡  (range 61-84) | 5/4 |  |  |  |  | x |  |  |  |  |  |  |  |  |  |  |  | Convenience | Interview (semi-structured) | Phenomenology | Decision-making process leading to opting out of dialysis and experience with conservative nondialytic management | |
| Tong, A.  2015^11^* | Australia | 41 | 35.6†  (range 22-56) | 0/41 |  |  | x | x | x |  |  | x | x |  |  | x |  |  |  |  | Purposive | Interview (semi-structured) | Thematic analysis | Beliefs, values, and experiences of pregnancy in women with CKD | |

**Table S1** Overview of the 20 studies with mixed populations (continued).

| DEMOGRAPHY | | | | | CKD PATIENTS  (stage) | | | | | | | RRT PATIENTS (type) | | | | | Others | | | | | RESEARCH METHODS | | |  |
| --- | --- | --- | --- | --- | --- | --- | --- | --- | --- | --- | --- | --- | --- | --- | --- | --- | --- | --- | --- | --- | --- | --- | --- | --- | --- |
| Study | Country | n. | Age | Sex m/f | CKD1 | CKD 2 | CKD3 | CKD4 | CKD5 | Predialysis | Unclear | HD | PD | CAPD | Unclear | RTx | Healthcare | Relatives | Children | Unknown | Sampling | Data gathering | Analysis | Principal research aim | **Study** |
| Kang, H.  2016^12^ | Canada | 53 | -  (range 19-70+) | 22/17 |  |  |  |  |  |  | x |  |  |  |  |  |  | x |  |  |  | Consecutive | Observations (unsolicited information) | Pierre Bourdieu’s relational | Tensions and gaps in understanding of “living well” with CKD and challenges in self-management and support. |
| Lederer, S.  2016^13^ | USA | 85 | 67†  (SD: 8.8) | 82/3 |  |  | x | x | X |  |  |  |  |  |  | x | x |  |  |  |  | Purposive | Interview (semi-structured) | Thematic analysis | Information needs to engage CKD patients in healthcare conversations |
| Walker, R. C.  2016^14^ | Australia/ New Zealand | 52 | 55†  (range 22-79) | 25/27 |  |  |  |  |  | x |  |  | x | x | x |  |  |  |  |  | x | Purposive | Interview (semi-structured) | Grounded theory | Values, beliefs and experiences when considering home dialysis |
| Lo, C.  2016^15^ | Australia | 58 | 67‡  (range 48-84) | 41/17 |  |  | x | x | x |  |  |  | x | x |  |  |  |  |  |  |  | Purposive | Focus group  Interview (semi-structured) | Thematic analysis (inductive) | Perspectives on factors influencing the health-care of patients with co-morbid diabetes and CKD |
| Loiselle, M. C.  2016^16^ | Canada | 32 | 62.3†  (range 20-84) | 14/18 |  |  |  |  |  | x |  |  | x | x |  | x |  | x | x |  | x | Purposive and convenience | Interview (semi-structured)  Focus group | Content analysis (deductive and inductive) | Decisional needs of patients with advanced CKD |
| Morris, R. L.  2016^17^ | United Kingdom | 40 | -  (range 20-60+) | 20/20 |  |  | x |  |  |  |  |  |  |  |  |  |  | x |  |  |  | Convenience  Purposive | Interview (semi-structured) | Content analysis (Normalisation Process Theory) | Processes that may enable or constrain the implementation of ‘sick day rules’ for AKI prevention |
| Subramanian, L.  2017^18^ | USA | 179 | 63.4†  - | 80/99 |  |  |  |  |  |  | x |  | x | x |  |  |  |  |  |  |  | Convenience | Interview (semi-structured) | Content analysis | Coping strategies, patients’ needs and healthcare providers’ support |
| Walker, R. C.  2017^19^ | New Zealand | 13 | 59†  (range 22-72) | - |  |  |  |  |  | x |  |  | x | x |  |  |  |  |  |  |  | Purposive | Interview (semi-structured) | Grounded theory and thematic analysis | Experiences and beliefs regarding CKD, and contextual, social and organisational factors |
| Smekal, M. D.  2018^20^ | Canada | 68 | -  (range 50-75+) | 26/42 |  |  |  |  |  |  | x |  |  |  |  |  |  | x | x |  |  | Convenience | Focus group  Interview (semi-structured) | Content analysis (inductive) | Perspectives of the perceived benefits and challenges of using a risk-based approach to guide care delivery |

These studies were used for analysis, but only those quotes or data that could be linked to a CKD patient not on dialysis, kidney transplant or conservative therapy. Data were pooled where possible: if age was given per subgroup, the mean or median age for the CKD subgroup is reported; if sex was given per subgroup, percentages were recalculated to absolute numbers and an overall male/female ratio is reported. Abbreviations: SD: standard deviation, CKD: Chronic Kidney Disease; FG: focus group; IV: interview; RRT: renal replacement therapy, RTx: renal transplantation, HD: haemodialysis; PD: peritoneal dialysis; CAPD: Continuous Ambulatory Peritoneal dialysis. *Studies with unspecified distribution CKD groups (e.g. "CKD 4-5 are marked as CKD 4 and CKD 5); +studies with eGFR ranges (e.g. "eGFR <30" are marked as CKD 4 and CKD 5) are attributed to a CKD stage according to the KDIGO CKD staging system; † mean; ‡ median.

**Table S2** Quality of reporting of the 46 included studies, per individual study, as assessed with the COREQ. If a topic is mentioned in the individual study, this is marked green; if not, it is marked red.

|  |  | **DOMAIN 1** | | | | | | | | **DOMAIN 2** | | | | | | | | | | | | | | | **DOMAIN 3** | | | | | | | | |
| --- | --- | --- | --- | --- | --- | --- | --- | --- | --- | --- | --- | --- | --- | --- | --- | --- | --- | --- | --- | --- | --- | --- | --- | --- | --- | --- | --- | --- | --- | --- | --- | --- | --- |
|  |  | Interviewer/facilitator | Credentials | Occupation | Gender | Experience and training | Relationship established | Participant knowledge of the interviewer | Interviewer characteristics | Methodological orientation and Theory | Sampling | Method of approach | Sample size | Non-participation | Setting of data collection | Presence of nonparticipants | Description of sample | Interview guide | Repeat interviews | Audio/visual recording | Field notes | Duration | Data saturation | Transcripts returned | Number of data coders | Description of the coding tree | Derivation of themes | Software | Participant checking | Quotations presented | Data and findings consistent | Clarity of major themes | Clarity of minor themes |
| Study | Score | 32/46 | 15/46 | 24/46 | 27/46 | 13/46 | 9/46 | 5/46 | 6/46 | 35/46 | 42/46 | 46/46 | 46/46 | 23/46 | 35/46 | 6/46 | 38/46 | 28/46 | 7/46 | 44/46 | 16/46 | 31/46 | 22/46 | 2/46 | 33/46 | 21/46 | 42/46 | 23/46 | 9/46 | 44/46 | 45/46 | 46/46 | 38/46 |
| Beard, B. H. 1969^1^ | 8/32 |  |  |  |  |  |  |  |  |  |  |  |  |  |  |  |  |  |  |  |  |  |  |  |  |  |  |  |  |  |  |  |  |
| Andrew, J. 2001^21^ | 14/32 |  |  |  |  |  |  |  |  |  |  |  |  |  |  |  |  |  |  |  |  |  |  |  |  |  |  |  |  |  |  |  |  |
| Iles-Smith, H. 2005^22^ | 16/32 |  |  |  |  |  |  |  |  |  |  |  |  |  |  |  |  |  |  |  |  |  |  |  |  |  |  |  |  |  |  |  |  |
| Tweed, A. E.2005^23^ | 19/32 |  |  |  |  |  |  |  |  |  |  |  |  |  |  |  |  |  |  |  |  |  |  |  |  |  |  |  |  |  |  |  |  |
| Costantini, L.2008^24^* | 15/32 |  |  |  |  |  |  |  |  |  |  |  |  |  |  |  |  |  |  |  |  |  |  |  |  |  |  |  |  |  |  |  |  |
| Tong, A.2008^2^ | 22/32 |  |  |  |  |  |  |  |  |  |  |  |  |  |  |  |  |  |  |  |  |  |  |  |  |  |  |  |  |  |  |  |  |
| Lee, A.2008^3^ | 17/32 |  |  |  |  |  |  |  |  |  |  |  |  |  |  |  |  |  |  |  |  |  |  |  |  |  |  |  |  |  |  |  |  |
| Sakraida, T. J.2009^25^ | 18/32 |  |  |  |  |  |  |  |  |  |  |  |  |  |  |  |  |  |  |  |  |  |  |  |  |  |  |  |  |  |  |  |  |
| Tong, A.2009^4^ | 17/32 |  |  |  |  |  |  |  |  |  |  |  |  |  |  |  |  |  |  |  |  |  |  |  |  |  |  |  |  |  |  |  |  |
| Noble, H.2010^26^ | 16/32 |  |  |  |  |  |  |  |  |  |  |  |  |  |  |  |  |  |  |  |  |  |  |  |  |  |  |  |  |  |  |  |  |
| de Brito-Ashurst, I.2011^27^ | 20/32 |  |  |  |  |  |  |  |  |  |  |  |  |  |  |  |  |  |  |  |  |  |  |  |  |  |  |  |  |  |  |  |  |
| Boulware, L. E.2011^5^ | 17/32 |  |  |  |  |  |  |  |  |  |  |  |  |  |  |  |  |  |  |  |  |  |  |  |  |  |  |  |  |  |  |  |  |
| Morton, R. L.2011^6^ | 19/32 |  |  |  |  |  |  |  |  |  |  |  |  |  |  |  |  |  |  |  |  |  |  |  |  |  |  |  |  |  |  |  |  |
| Nygardh, A.2012^28^ | 20/32 |  |  |  |  |  |  |  |  |  |  |  |  |  |  |  |  |  |  |  |  |  |  |  |  |  |  |  |  |  |  |  |  |
| Sakraida, T. J.2012^29^ | 25/32 |  |  |  |  |  |  |  |  |  |  |  |  |  |  |  |  |  |  |  |  |  |  |  |  |  |  |  |  |  |  |  |  |
| Walker, R.2012^30^+ | 15/32 |  |  |  |  |  |  |  |  |  |  |  |  |  |  |  |  |  |  |  |  |  |  |  |  |  |  |  |  |  |  |  |  |
| Johnston, S.2012^31^ | 17/32 |  |  |  |  |  |  |  |  |  |  |  |  |  |  |  |  |  |  |  |  |  |  |  |  |  |  |  |  |  |  |  |  |
| McKillop, G.2013^32^ | 15/32 |  |  |  |  |  |  |  |  |  |  |  |  |  |  |  |  |  |  |  |  |  |  |  |  |  |  |  |  |  |  |  |  |
| Lin, C. C.2013^33^* | 20/32 |  |  |  |  |  |  |  |  |  |  |  |  |  |  |  |  |  |  |  |  |  |  |  |  |  |  |  |  |  |  |  |  |
| Llewellyn, H.2014^7^ | 17/32 |  |  |  |  |  |  |  |  |  |  |  |  |  |  |  |  |  |  |  |  |  |  |  |  |  |  |  |  |  |  |  |  |
| Lopez-Vargas, P. A.2014^34^ | 23/32 |  |  |  |  |  |  |  |  |  |  |  |  |  |  |  |  |  |  |  |  |  |  |  |  |  |  |  |  |  |  |  |  |
| Tangkiatkumjai, M.2014^35^* | 10/32 |  |  |  |  |  |  |  |  |  |  |  |  |  |  |  |  |  |  |  |  |  |  |  |  |  |  |  |  |  |  |  |  |
| Meuleman, Y.2014^8^ | 17/32 |  |  |  |  |  |  |  |  |  |  |  |  |  |  |  |  |  |  |  |  |  |  |  |  |  |  |  |  |  |  |  |  |
| Clarke, A. L.2015^36^ | 17/32 |  |  |  |  |  |  |  |  |  |  |  |  |  |  |  |  |  |  |  |  |  |  |  |  |  |  |  |  |  |  |  |  |
| Erlang, A. S.2015^37^ | 15/32 |  |  |  |  |  |  |  |  |  |  |  |  |  |  |  |  |  |  |  |  |  |  |  |  |  |  |  |  |  |  |  |  |
| Lederer, S.2015^9^ | 23/32 |  |  |  |  |  |  |  |  |  |  |  |  |  |  |  |  |  |  |  |  |  |  |  |  |  |  |  |  |  |  |  |  |

**Table S2 (continued)** quality of reporting of the 46 included studies, per individual study, as assessed with the COREQ. If a topic is mentioned in the individual study, this is marked green; if not, it is marked red.

|  |  | **DOMAIN 1** | | | | | | | | **DOMAIN 2** | | | | | | | | | | | | | | | **DOMAIN 3** | | | | | | | | |
| --- | --- | --- | --- | --- | --- | --- | --- | --- | --- | --- | --- | --- | --- | --- | --- | --- | --- | --- | --- | --- | --- | --- | --- | --- | --- | --- | --- | --- | --- | --- | --- | --- | --- |
|  |  | Interviewer/facilitator | Credentials | Occupation | Gender | Experience and training | Relationship established | Participant knowledge of the interviewer | Interviewer characteristics | Methodological orientation and Theory | Sampling | Method of approach | Sample size | Non-participation | Setting of data collection | Presence of nonparticipants | Description of sample | Interview guide | Repeat interviews | Audio/visual recording | Field notes | Duration | Data saturation | Transcripts returned | Number of data coders | Description of the coding tree | Derivation of themes | Software | Participant checking | Quotations presented | Data and findings consistent | Clarity of major themes | Clarity of minor themes |
| Study | Score | 32/46 | 15/46 | 24/46 | 27/46 | 13/46 | 9/46 | 5/46 | 6/46 | 35/46 | 42/46 | 46/46 | 46/46 | 23/46 | 35/46 | 6/46 | 38/46 | 28/46 | 7/46 | 44/46 | 16/46 | 32/46 | 22/46 | 2/46 | 33/46 | 21/46 | 42/46 | 23/46 | 9/46 | 44/46 | 45/46 | 46/46 | 38/46 |
| Seah, A. S.2015^10^ | 20/32 |  |  |  |  |  |  |  |  |  |  |  |  |  |  |  |  |  |  |  |  |  |  |  |  |  |  |  |  |  |  |  |  |
| Tong, A.2015^11^* | 20/32 |  |  |  |  |  |  |  |  |  |  |  |  |  |  |  |  |  |  |  |  |  |  |  |  |  |  |  |  |  |  |  |  |
| Kang, H.2016^12^ | 19/32 |  |  |  |  |  |  |  |  |  |  |  |  |  |  |  |  |  |  |  |  |  |  |  |  |  |  |  |  |  |  |  |  |
| Lederer, S.2016^13^ | 22/32 |  |  |  |  |  |  |  |  |  |  |  |  |  |  |  |  |  |  |  |  |  |  |  |  |  |  |  |  |  |  |  |  |
| Shirazian, S.2016^38^* | 25/32 |  |  |  |  |  |  |  |  |  |  |  |  |  |  |  |  |  |  |  |  |  |  |  |  |  |  |  |  |  |  |  |  |
| Walker, R. C.2016^14^ | 26/32 |  |  |  |  |  |  |  |  |  |  |  |  |  |  |  |  |  |  |  |  |  |  |  |  |  |  |  |  |  |  |  |  |
| Wright Nunes, J.2016^39^* | 17/32 |  |  |  |  |  |  |  |  |  |  |  |  |  |  |  |  |  |  |  |  |  |  |  |  |  |  |  |  |  |  |  |  |
| Wu, C. C.2016^40^* | 15/32 |  |  |  |  |  |  |  |  |  |  |  |  |  |  |  |  |  |  |  |  |  |  |  |  |  |  |  |  |  |  |  |  |
| Lo, C.2016^15^ | 19/32 |  |  |  |  |  |  |  |  |  |  |  |  |  |  |  |  |  |  |  |  |  |  |  |  |  |  |  |  |  |  |  |  |
| Loiselle, M. C.2016^16^ | 20/32 |  |  |  |  |  |  |  |  |  |  |  |  |  |  |  |  |  |  |  |  |  |  |  |  |  |  |  |  |  |  |  |  |
| Morris, R. L.2016^17^ | 15/32 |  |  |  |  |  |  |  |  |  |  |  |  |  |  |  |  |  |  |  |  |  |  |  |  |  |  |  |  |  |  |  |  |
| Schipper, K.2016^41^* | 19/32 |  |  |  |  |  |  |  |  |  |  |  |  |  |  |  |  |  |  |  |  |  |  |  |  |  |  |  |  |  |  |  |  |
| Bowling, C. B.2017^42^+ | 19/32 |  |  |  |  |  |  |  |  |  |  |  |  |  |  |  |  |  |  |  |  |  |  |  |  |  |  |  |  |  |  |  |  |
| Havas, K.2017^43^* | 22/32 |  |  |  |  |  |  |  |  |  |  |  |  |  |  |  |  |  |  |  |  |  |  |  |  |  |  |  |  |  |  |  |  |
| Subramanian, L.2017^18^ | 17/32 |  |  |  |  |  |  |  |  |  |  |  |  |  |  |  |  |  |  |  |  |  |  |  |  |  |  |  |  |  |  |  |  |
| Walker, R. C.2017^19^ | 27/32 |  |  |  |  |  |  |  |  |  |  |  |  |  |  |  |  |  |  |  |  |  |  |  |  |  |  |  |  |  |  |  |  |
| Lovell, S.2017^44^ | 22/32 |  |  |  |  |  |  |  |  |  |  |  |  |  |  |  |  |  |  |  |  |  |  |  |  |  |  |  |  |  |  |  |  |
| Pugh-Clarke, K.2017^45^* | 15/32 |  |  |  |  |  |  |  |  |  |  |  |  |  |  |  |  |  |  |  |  |  |  |  |  |  |  |  |  |  |  |  |  |
| Campbell-Crofts, S.2018^46^* | 22/32 |  |  |  |  |  |  |  |  |  |  |  |  |  |  |  |  |  |  |  |  |  |  |  |  |  |  |  |  |  |  |  |  |
| Smekal, M. D.2018^20^ | 21/32 |  |  |  |  |  |  |  |  |  |  |  |  |  |  |  |  |  |  |  |  |  |  |  |  |  |  |  |  |  |  |  |  |

**Table S3** Overview of symptoms mentioned in the included 46 articles. Using a deductive approach, symptoms were grouped into categories. In some cases, a more general term is used to describe certain symptoms for brevity, e.g. ‘appetite’ was used to describe increased or decreased appetite; ‘skin’ was used for dry, weak or brittle skin, etc.

|  |  | **CKD ONLY** | | | | | | | | | | | | | | | | | | | | | | | | | | | **MIXED** | | | | | | | | | | | | | | | | | | | |
| --- | --- | --- | --- | --- | --- | --- | --- | --- | --- | --- | --- | --- | --- | --- | --- | --- | --- | --- | --- | --- | --- | --- | --- | --- | --- | --- | --- | --- | --- | --- | --- | --- | --- | --- | --- | --- | --- | --- | --- | --- | --- | --- | --- | --- | --- | --- | --- | --- |
|  |  | | **Andrew, J. 2001^21^** | **Iles-Smith, H. 2005^22^** | **Tweed, A. E.2005^23^** | **Costantini, L.2008^24^** | **Sakraida, T. J.2009^25^** | **Noble, H.2010^26^** | **de Brito-Ashurst, I.2011^27^** | **Johnston, S.2012^31^** | **Sakraida, T. J.2012^29^** | **Walker, R.2012^30^** | **Nygardh, A.2012^28^** | **Lin, C. C.2013^33^** | **McKillop, G.2013^32^** | **Lopez-Vargas, P. A.2014^34^** | **Tangkiatkumjai, M.2014^35^** | **Clarke, A. L.2015^36^** | **Erlang, A. S.2015^37^** | **Schipper, K.2016^41^** | **Shirazian, S.2016^38^** | **Wright Nunes, J.2016^39^** | **Wu, C. C.2016^40^** | **Bowling, C. B.2017^42^** | **Havas, K.2017^43^** | **Lovell, S.2017^44^** | **Pugh-Clarke, K.2017^45^** | **Campbell-Crofts, S.2018^46^** | **Beard, B. H. 1969^1^** | **Tong, A.2008^2^** | **Lee, A.2008^3^** | **Tong, A.2009^4^** | **Boulware, L. E.2011^5^** | **Morton, R. L.2011^6^** | **Llewellyn, H.2014^7^** | **Meuleman, Y.2014^8^** | **Lederer, S.2015^9^** | **Seah, A. S.2015^10^** | **Tong, A.2015^11^** | **Lo, C.2016^15^** | **Kang, H.2016^12^** | **Lederer, S.2016^13^** | **Loiselle, M. C.2016^16^** | **Morris, R. L.2016^17^** | **Walker, R. C.2016^14^** | **Subramanian, L.2017^18^** | **Walker, R. C.2017^19^** | **Smekal, M. D.2018^20^** |
| Digestive | Constipation | |  |  |  |  |  | x |  |  |  |  |  |  |  |  |  |  |  |  |  |  |  |  |  |  | x |  |  |  |  |  |  |  |  |  |  |  |  |  |  |  |  |  |  |  |  |  |
|  | Diarrhoea | |  |  |  |  |  | x |  |  |  |  |  |  |  |  |  |  |  |  |  |  |  |  |  |  | x |  |  |  |  |  |  |  |  |  |  |  |  |  |  |  |  |  |  |  |  |  |
|  | Nausea | |  |  |  |  |  | x |  |  |  |  |  |  | x |  |  |  |  |  |  |  |  |  |  |  | x |  |  | x |  | x |  |  |  |  |  | x |  |  |  |  |  |  |  |  |  |  |
|  | Poor appetite | |  |  |  |  |  | x |  |  |  |  |  |  |  |  |  |  |  |  |  |  |  |  |  |  |  |  |  |  |  |  |  |  |  |  |  |  |  |  |  |  |  |  |  |  |  |  |
|  | Vomiting | |  |  |  |  |  | x |  |  |  |  |  |  |  |  |  |  |  |  |  |  |  |  |  |  |  |  |  | x |  | x |  |  |  |  |  |  |  |  |  |  |  |  |  |  |  |  |
|  | Faecal incontinence | |  |  |  |  |  | x |  |  |  |  |  |  |  |  |  |  |  |  |  |  |  |  |  |  |  |  |  |  |  |  |  |  |  |  |  |  |  |  |  |  |  |  |  |  |  |  |
|  | Appetite | |  |  |  |  |  |  |  |  |  |  |  |  |  |  |  |  |  |  |  |  |  |  |  | x |  |  |  |  |  |  |  |  |  |  |  |  |  |  |  |  |  |  |  |  |  |  |
|  | Anorexia / weight loss | |  |  |  |  |  | x |  |  |  |  |  |  |  |  |  |  |  |  |  |  |  |  |  |  | x |  |  |  |  |  |  |  |  |  |  |  |  |  | x |  |  |  |  |  |  |  |
|  | Weight increase | |  |  |  |  |  |  |  |  |  |  |  |  |  |  |  |  |  |  |  |  | x |  |  |  |  |  |  |  |  |  |  |  |  |  |  |  |  |  |  |  |  |  |  |  |  |  |
|  | Indigestion | |  |  |  |  |  |  |  |  |  |  |  |  |  |  |  |  |  |  |  |  |  |  |  |  | x |  |  |  |  |  |  |  |  |  |  |  |  |  |  |  |  |  |  |  |  |  |
|  | Flatulence | |  |  |  |  |  |  |  |  |  |  |  |  |  |  | x |  |  |  |  |  |  |  |  |  |  |  |  |  |  |  |  |  |  |  |  |  |  |  |  |  |  |  |  |  |  |  |
| Cardiovascular | Puffiness (oedema) | |  |  |  |  |  | x |  |  |  |  |  | x |  |  |  |  |  |  |  | x | x |  |  |  | x |  |  |  |  |  |  |  |  |  |  |  |  |  |  |  |  |  |  |  |  |  |
|  | Orthopnoea | |  |  |  |  |  |  |  |  |  |  |  |  |  |  |  |  |  |  |  |  |  |  |  |  | x |  |  |  |  |  |  |  |  |  |  |  |  |  |  |  |  |  |  |  |  |  |
|  | Chest pain (angina) | |  |  |  |  |  |  |  |  |  |  |  |  |  |  |  |  |  |  |  |  |  |  |  |  | x |  |  |  |  |  |  |  |  |  |  | x |  |  |  |  |  |  |  |  |  |  |
|  | Hypertension | |  |  |  |  |  |  |  |  |  |  |  |  |  |  |  |  |  |  |  |  |  |  |  |  |  |  |  |  |  |  | x |  |  |  |  |  |  |  |  | x |  |  |  |  |  |  |
| Pulmonary | Dyspnoea | |  |  |  |  |  | x |  |  |  |  |  |  |  |  |  | x |  |  |  |  | x |  |  |  |  |  |  |  |  |  |  |  | x |  |  |  |  |  |  |  |  |  |  |  |  |  |
|  | Hiccups | |  |  |  |  |  |  |  |  |  |  |  |  |  |  |  |  |  |  |  |  |  |  |  |  | x |  |  |  |  |  |  |  |  |  |  |  |  |  |  |  |  |  |  |  |  |  |
| Urogenital | Kidney or flank pain | |  |  |  |  |  |  |  |  |  |  |  | x |  |  |  |  |  | x |  |  |  |  |  |  | x |  |  |  |  |  |  |  |  |  |  |  |  |  |  |  |  |  |  |  |  |  |
|  | Foamy urine | |  |  |  |  |  |  |  |  |  |  |  | x |  |  |  |  |  |  |  |  | x |  |  |  |  |  |  |  |  |  |  |  |  |  |  |  |  |  |  |  |  |  |  |  |  |  |
|  | Incontinence | |  |  |  |  |  | x |  |  |  |  |  |  |  |  |  |  |  |  |  |  |  |  |  |  | x |  |  |  |  |  |  |  |  |  |  |  |  |  |  |  |  |  |  |  |  |  |
|  | Nycturia | |  |  |  |  |  |  |  |  |  |  |  |  |  |  |  |  |  |  |  |  |  |  |  |  | x |  |  |  |  |  |  |  |  |  |  |  |  |  |  |  |  |  |  |  |  |  |
|  | Polyuria | |  |  |  |  |  |  |  |  |  |  |  |  |  |  |  |  |  |  |  |  | x |  |  |  | x |  |  |  |  |  |  |  |  |  |  |  |  |  |  |  |  |  |  |  |  |  |
|  | Urinary urgency | |  |  |  |  |  |  |  |  |  |  |  |  |  |  |  |  |  |  |  |  |  |  |  |  | x |  |  |  |  |  |  |  |  |  |  |  |  |  |  |  |  |  |  |  |  |  |
|  | Oliguria | |  |  |  |  |  |  |  |  |  |  |  |  |  |  |  |  |  |  |  |  |  |  |  |  | x |  |  |  |  |  |  |  |  |  |  |  |  |  |  |  |  |  |  |  |  |  |
|  | Dysuria | |  |  |  |  |  |  |  |  |  |  |  |  |  |  |  |  |  |  |  |  |  |  |  |  | x |  |  |  |  |  |  |  |  |  |  |  |  |  |  |  |  |  |  |  |  |  |
|  | Unspecified | |  |  |  |  |  |  |  |  |  |  |  |  |  |  |  |  |  |  |  |  |  |  |  |  |  |  |  |  |  |  |  |  | x |  |  |  |  |  |  |  |  |  |  |  |  |  |

**Table S3** (continued) Overview of symptoms mentioned in the included 46 articles.

|  | |  | | **CKD ONLY** | | | | | | | | | | | | | | | | | | | | | | | | | | | **MIXED** | | | | | | | | | | | | | | | | | | | |
| --- | --- | --- | --- | --- | --- | --- | --- | --- | --- | --- | --- | --- | --- | --- | --- | --- | --- | --- | --- | --- | --- | --- | --- | --- | --- | --- | --- | --- | --- | --- | --- | --- | --- | --- | --- | --- | --- | --- | --- | --- | --- | --- | --- | --- | --- | --- | --- | --- | --- | --- |
|  | |  | | | **Andrew, J. 2001^21^** | **Iles-Smith, H. 2005^22^** | **Tweed, A. E.2005^23^** | **Costantini, L.2008^24^** | **Sakraida, T. J.2009^25^** | **Noble, H.2010^26^** | **de Brito-Ashurst, I.2011^27^** | **Johnston, S.2012^31^** | **Sakraida, T. J.2012^29^** | **Walker, R.2012^30^** | **Nygardh, A.2012^28^** | **Lin, C. C.2013^33^** | **McKillop, G.2013^32^** | **Lopez-Vargas, P. A.2014^34^** | **Tangkiatkumjai, M.2014^35^** | **Clarke, A. L.2015^36^** | **Erlang, A. S.2015^37^** | **Schipper, K.2016^41^** | **Shirazian, S.2016^38^** | **Wright Nunes, J.2016^39^** | **Wu, C. C.2016^40^** | **Bowling, C. B.2017^42^** | **Havas, K.2017^43^** | **Lovell, S.2017^44^** | **Pugh-Clarke, K.2017^45^** | **Campbell-Crofts, S.2018^46^** | **Beard, B. H. 1969^1^** | **Tong, A.2008^2^** | **Lee, A.2008^3^** | **Tong, A.2009^4^** | **Boulware, L. E.2011^5^** | **Morton, R. L.2011^6^** | **Llewellyn, H.2014^7^** | **Meuleman, Y.2014^8^** | **Lederer, S.2015^9^** | **Seah, A. S.2015^10^** | **Tong, A.2015^11^** | **Lo, C.2016^15^** | **Kang, H.2016^12^** | **Lederer, S.2016^13^** | **Loiselle, M. C.2016^16^** | **Morris, R. L.2016^17^** | **Walker, R. C.2016^14^** | **Subramanian, L.2017^18^** | **Walker, R. C.2017^19^** | **Smekal, M. D.2018^20^** |
| Neurological | | Dizziness | |  |  |  |  |  | x |  |  |  |  |  | x |  |  | x |  |  |  |  |  | x |  |  |  | x |  |  |  |  |  |  |  |  |  |  |  |  |  |  |  |  |  |  |  |  |  |  |
|  | | Headaches | |  |  |  |  |  | x |  |  |  |  |  |  |  |  |  |  |  |  |  |  |  |  |  |  | x |  |  |  |  |  |  |  |  |  |  | x |  |  |  |  |  |  |  |  |  |  |  |
|  | | Forgetfulness | |  |  |  |  |  | x |  |  |  |  |  | x | x |  |  |  |  | x |  |  |  |  |  | x |  |  |  |  |  |  |  |  |  |  |  |  |  |  |  |  |  |  |  |  |  |  |  |
|  | | Confusion | |  |  |  |  |  | x |  |  |  |  |  |  |  |  |  |  |  |  |  |  |  |  |  |  |  |  |  |  |  |  |  |  |  |  |  |  |  |  |  |  |  |  |  |  |  |  |  |
|  | | Poor balance | |  |  |  |  |  | x |  |  |  |  |  |  |  |  |  |  |  |  |  |  |  |  |  |  |  |  |  |  |  |  |  |  |  |  |  |  |  |  |  |  |  |  |  |  |  |  |  |
|  | | Smell | |  |  |  |  |  |  |  |  |  |  |  |  |  |  |  |  |  |  |  |  |  |  |  |  | x |  |  |  |  |  |  |  |  |  |  |  |  |  |  |  |  |  |  |  |  |  |  |
|  | | Taste | |  |  |  |  |  | x |  |  |  |  |  |  |  |  |  |  |  |  |  |  |  |  |  |  | x |  |  |  |  |  |  |  |  |  |  |  |  |  |  |  |  |  |  |  |  |  |  |
|  | | Poor vision | |  |  |  |  |  | x |  |  |  |  |  |  |  |  |  |  |  |  |  |  |  |  |  |  |  |  |  |  |  |  |  |  |  |  |  |  |  |  |  |  |  |  |  |  |  |  |  |
|  | | Balance disorders | |  |  |  |  |  | x |  |  |  |  |  |  |  |  |  |  |  |  |  |  |  |  |  |  | x |  |  |  |  |  |  |  |  |  |  |  |  |  |  |  |  |  |  |  |  |  |  |
|  | | Collapse | |  |  |  |  |  |  |  |  |  |  |  |  |  |  |  |  |  |  |  |  |  |  |  |  | x |  |  |  |  |  |  |  |  |  |  |  |  |  |  |  |  |  |  |  |  |  |  |
|  | | Tremor | |  |  |  |  |  | x |  |  |  |  |  |  |  |  |  |  |  |  |  |  |  |  |  |  | x |  |  |  |  |  |  |  |  |  |  |  |  |  |  |  |  |  |  |  |  |  |  |
| Psychological | | Depression | |  |  |  |  |  | x |  |  |  |  |  |  |  |  |  |  |  |  |  |  |  |  |  |  | x |  | x |  |  | x |  |  | x |  |  |  |  | x |  |  |  |  |  |  |  |  |  |
|  | | Insomnia | |  |  |  |  |  | x |  |  |  |  |  |  |  |  |  |  |  |  |  |  |  |  |  |  | x |  |  | x |  |  |  |  |  |  |  |  |  |  |  |  |  |  |  |  |  |  |  |
|  | | Lethargy | |  |  |  |  |  | x |  |  |  |  |  |  |  |  |  |  |  |  |  |  |  |  |  |  | x |  |  |  |  |  |  |  |  |  |  |  |  |  |  |  |  |  |  |  |  |  |  |
|  | | Stress or anxiety | |  |  |  |  | x |  |  |  |  |  |  |  |  |  |  |  |  |  |  |  |  |  | x |  |  |  | x |  |  | x |  |  |  |  |  |  | x |  | x |  | x |  |  |  |  |  |  |
|  | | Weariness | |  |  |  |  |  |  |  |  |  |  |  |  |  |  |  |  |  |  |  |  |  |  |  |  | x |  |  |  |  |  |  |  |  |  |  |  |  |  |  |  |  |  |  |  |  |  |  |
|  | | Undefined | |  |  |  |  |  |  |  |  |  |  |  |  |  |  |  |  |  |  |  |  |  |  |  |  |  |  |  | x |  |  |  |  |  |  |  |  |  |  |  |  |  |  |  | x |  |  |  |
| Ophthalmic | | Dry eyes | |  |  |  |  |  |  |  |  |  |  |  |  |  |  |  |  |  |  |  |  |  |  |  |  | x |  |  |  |  |  |  |  |  |  |  |  |  |  |  |  |  |  |  |  |  |  |  |
|  | | Vision | |  |  |  |  |  |  |  |  |  |  |  |  |  |  |  |  |  |  |  |  |  |  |  |  | x |  |  |  |  |  |  |  |  |  |  |  |  |  |  |  |  |  |  |  |  |  |  |
|  | | Sore or itchy eyes | |  |  |  |  |  |  |  |  |  |  |  |  |  |  |  |  |  |  |  |  |  |  |  |  | x |  |  |  |  |  |  |  |  |  |  |  |  |  |  |  |  |  |  |  |  |  |  |
| Immunology/ | | Recurrent infections | |  |  |  |  |  |  |  |  |  |  |  |  |  |  |  |  |  |  |  |  | x |  |  |  | x |  |  |  |  |  |  |  | x |  |  |  |  |  |  |  |  |  |  |  |  |  |  |
| haematology | | Anaemia | |  |  |  |  |  |  |  |  |  |  |  |  |  |  |  |  |  |  |  |  | x |  |  |  |  |  |  |  |  |  |  |  |  |  | x |  |  |  |  | x |  |  |  |  |  |  |  |
| Sexual | | Genital dysfunction | |  |  |  |  |  |  |  |  |  | x |  |  |  |  |  |  |  | x |  |  |  |  |  |  | x |  |  |  |  |  |  |  |  |  |  |  |  |  |  |  |  |  |  |  |  |  |  |
|  | | Infertility | |  |  |  |  |  |  |  |  |  |  |  |  |  |  |  |  |  | x |  |  |  |  |  |  |  |  | x |  |  |  |  |  |  |  |  |  | x |  |  |  |  |  |  |  |  |  |  |
|  | | Loss of libido | |  |  |  |  |  |  |  |  |  | x |  |  |  |  |  |  |  | x |  |  |  |  |  |  | x |  |  |  |  |  |  |  |  |  |  |  |  |  |  |  |  |  |  |  |  |  |  |
|  | |  | |  |  |  |  |  |  |  |  |  |  |  |  |  |  |  |  |  |  |  |  |  |  |  |  |  |  |  |  |  |  |  |  |  |  |  |  |  |  |  |  |  |  |  |  |  |  |  |

**Table S3** (continued) Overview of symptoms mentioned in the included 46 articles.

|  | |  | **CKD ONLY** | | | | | | | | | | | | | | | | | | | | | | | | | | | | | | | | | | | | | | | | | **MIXED** | | | | | | | | | | | | | | | | | | | |
| --- | --- | --- | --- | --- | --- | --- | --- | --- | --- | --- | --- | --- | --- | --- | --- | --- | --- | --- | --- | --- | --- | --- | --- | --- | --- | --- | --- | --- | --- | --- | --- | --- | --- | --- | --- | --- | --- | --- | --- | --- | --- | --- | --- | --- | --- | --- | --- | --- | --- | --- | --- | --- | --- | --- | --- | --- | --- | --- | --- | --- | --- | --- | --- |
|  | |  | | | **Andrew, J. 2001^21^** | | **Iles-Smith, H. 2005^22^** | | **Tweed, A. E.2005^23^** | | **Costantini, L.2008^24^** | | **Sakraida, T. J.2009^25^** | | **Noble, H.2010^26^** | | **de Brito-Ashurst, I.2011^27^** | | **Johnston, S.2012^31^** | | **Sakraida, T. J.2012^29^** | | **Walker, R.2012^30^** | | **Nygardh, A.2012^28^** | | **Lin, C. C.2013^33^** | | **McKillop, G.2013^32^** | | **Lopez-Vargas, P. A.2014^34^** | **Tangkiatkumjai, M.2014^35^** | **Clarke, A. L.2015^36^** | **Erlang, A. S.2015^37^** | **Schipper, K.2016^41^** | **Shirazian, S.2016^38^** | **Wright Nunes, J.2016^39^** | **Wu, C. C.2016^40^** | **Bowling, C. B.2017^42^** | **Havas, K.2017^43^** | **Lovell, S.2017^44^** | **Pugh-Clarke, K.2017^45^** | **Campbell-Crofts, S.2018^46^** | **Beard, B. H. 1969^1^** | **Tong, A.2008^2^** | **Lee, A.2008^3^** | **Tong, A.2009^4^** | **Boulware, L. E.2011^5^** | **Morton, R. L.2011^6^** | **Llewellyn, H.2014^7^** | **Meuleman, Y.2014^8^** | **Lederer, S.2015^9^** | **Seah, A. S.2015^10^** | **Tong, A.2015^11^** | **Lo, C.2016^15^** | **Kang, H.2016^12^** | **Lederer, S.2016^13^** | **Loiselle, M. C.2016^16^** | **Morris, R. L.2016^17^** | **Walker, R. C.2016^14^** | **Subramanian, L.2017^18^** | **Walker, R. C.2017^19^** | **Smekal, M. D.2018^20^** |
| Locomotorial | Cramps | | |  | |  | |  | |  | |  | | x | |  | |  | |  | |  | |  | |  | |  | |  | |  |  |  | x |  |  |  |  |  |  | x |  |  | x |  |  |  |  |  |  |  |  |  |  |  |  |  |  |  |  |  |  |
|  | Immobility | | |  | |  | |  | |  | |  | | x | |  | |  | |  | |  | |  | |  | |  | | x | |  |  |  |  |  |  |  |  |  |  | x |  |  |  |  |  |  | x |  |  |  |  |  | x |  |  |  |  |  |  |  |  |
|  | Joint problems | | |  | |  | |  | |  | |  | |  | |  | |  | |  | |  | |  | | x | |  | |  | |  | x |  |  |  |  |  |  |  |  | x |  |  |  |  |  |  |  |  |  |  |  |  |  |  |  |  |  |  |  |  |  |
|  | Aching body | | |  | |  | |  | |  | |  | | x | |  | |  | |  | |  | |  | |  | |  | |  | |  |  |  |  |  |  |  |  |  |  |  |  |  |  |  |  |  |  |  |  |  | x |  |  |  |  |  |  |  |  |  |  |
|  | Muscle weakness | | |  | |  | |  | |  | |  | |  | |  | |  | |  | |  | |  | |  | |  | |  | |  |  |  |  |  |  |  |  |  |  | x |  |  |  |  |  |  |  |  |  |  |  |  |  |  |  |  |  |  |  |  |  |
|  | Bone | | |  | |  | |  | |  | |  | |  | |  | |  | |  | |  | |  | |  | |  | |  | |  |  |  |  |  |  |  |  |  |  | x |  |  | x |  |  |  |  |  |  | x |  |  |  |  | x |  |  |  |  |  |  |
|  | Back pain | | |  | |  | |  | |  | |  | |  | |  | |  | |  | |  | |  | |  | |  | |  | |  |  |  |  |  |  |  |  |  |  | x |  |  |  |  |  |  |  |  |  |  |  |  |  |  |  |  |  |  |  |  |  |
|  | Gout | | |  | |  | |  | |  | |  | |  | |  | |  | |  | |  | |  | |  | |  | |  | |  |  |  | x |  |  |  |  |  |  |  |  |  |  |  |  |  |  |  |  |  |  |  |  |  |  |  |  |  |  |  |  |
| Eye Nose Troat (ENT) | Swallowing | | |  | |  | |  | |  | |  | | x | |  | |  | |  | |  | |  | |  | |  | |  | |  |  |  |  |  |  |  |  |  |  |  |  |  |  |  |  |  |  |  |  |  |  |  |  |  |  |  |  |  |  |  |  |
|  | Hearing | | |  | |  | |  | |  | |  | |  | |  | |  | |  | |  | |  | |  | |  | |  | |  |  |  |  |  |  |  |  |  | x |  |  |  |  |  |  |  |  |  |  |  |  |  |  |  |  |  |  |  |  |  |  |
|  | Sore mouth or throat | | |  | |  | |  | |  | |  | | x | |  | |  | |  | |  | |  | |  | |  | |  | |  |  |  |  |  |  |  |  |  |  | x |  |  |  |  |  |  |  |  |  |  |  |  |  |  |  |  |  |  |  |  |  |
|  | Dry mouth | | |  | |  | |  | |  | |  | |  | |  | |  | |  | |  | |  | |  | |  | |  | |  |  |  |  |  |  |  |  |  |  | x |  |  |  |  |  |  |  |  |  |  |  |  |  |  |  |  |  |  |  |  |  |
| Skin, hair and nails | Nails | | |  | |  | |  | |  | |  | |  | |  | |  | |  | |  | |  | |  | |  | |  | |  |  |  |  |  |  |  |  |  |  | x |  |  |  |  |  |  |  |  |  |  |  |  |  |  |  |  |  |  |  |  |  |
|  | Hair | | |  | |  | |  | |  | |  | |  | |  | |  | |  | |  | |  | |  | |  | |  | |  |  |  |  |  |  |  |  |  |  | x |  |  |  |  |  |  |  |  |  |  |  |  |  |  |  |  |  |  |  |  |  |
|  | Skin | | |  | |  | |  | |  | |  | | x | |  | |  | |  | |  | |  | |  | |  | |  | |  |  |  |  |  |  | x |  |  |  | x |  |  |  |  |  |  |  |  |  |  |  |  |  |  |  |  |  |  |  |  |  |
| Other | Fatigue | | |  | |  | |  | |  | |  | | x | |  | |  | |  | | x | |  | | x | | x | |  | |  | x |  | x |  |  | x |  | x | x | x |  |  | x |  | x |  | x |  |  |  |  |  | x | x |  |  |  |  |  |  |  |
|  | Feeling dry | | |  | |  | |  | |  | |  | | x | |  | |  | |  | |  | |  | |  | |  | |  | |  |  |  |  |  |  |  |  | x |  |  |  |  |  |  |  |  |  |  |  |  |  |  |  |  |  |  |  |  |  |  |  |
|  | General weakness or malaise | | |  | |  | |  | |  | |  | | x | |  | |  | |  | | x | |  | | x | | x | |  | |  |  |  |  |  |  |  |  |  |  | x |  |  | x |  |  |  |  | x |  |  | x |  | x | x |  |  |  |  |  |  |  |
|  | Pain | | |  | |  | |  | |  | |  | | x | |  | |  | |  | |  | |  | |  | |  | | x | |  |  |  |  | x |  |  |  |  |  |  |  |  |  |  |  |  |  | x |  |  |  |  |  |  |  |  |  |  |  |  |  |
|  | Phantom pain | | |  | |  | |  | |  | |  | | x | |  | |  | |  | |  | |  | |  | |  | |  | |  |  |  |  |  |  |  |  |  |  |  |  |  |  |  |  |  |  |  |  |  |  |  |  |  |  |  |  |  |  |  |  |
|  | Pins and needles in legs | | |  | |  | |  | |  | |  | | x | |  | |  | |  | |  | |  | |  | |  | |  | |  |  |  |  |  |  |  |  |  |  |  |  |  |  |  |  |  |  |  |  |  |  |  |  |  |  |  |  |  |  |  |  |
|  | Pruritus | | |  | |  | |  | |  | |  | | x | |  | |  | |  | |  | |  | |  | |  | |  | |  |  |  | x |  |  |  |  |  |  | x |  |  |  |  |  |  |  |  |  |  |  |  |  |  |  |  |  |  |  |  |  |
|  | Restless legs | | |  | |  | |  | |  | |  | |  | |  | |  | |  | |  | |  | |  | |  | |  | |  |  |  | x |  |  |  |  |  |  | x |  |  | x |  | x |  |  |  |  |  |  |  |  | x |  |  |  |  |  |  |  |
|  | Sleeplessness | | |  | |  | |  | |  | |  | | x | |  | |  | |  | |  | |  | |  | |  | |  | |  |  |  |  |  |  |  |  |  |  |  |  |  |  |  |  |  |  |  |  |  |  |  |  |  |  |  |  |  |  |  |  |
|  | Temperature sensitivity | | |  | |  | |  | |  | |  | |  | |  | |  | |  | |  | |  | |  | |  | |  | |  |  |  | x |  |  |  |  |  |  | x |  |  |  |  |  |  |  |  |  |  |  |  |  |  |  |  |  |  |  |  |  |
|  | Night sweats | | |  | |  | |  | |  | |  | |  | |  | |  | |  | |  | |  | |  | |  | |  | |  |  |  |  |  |  |  |  |  |  | x |  |  |  |  |  |  |  |  |  |  |  |  |  |  |  |  |  |  |  |  |  |
|  |  | | |  | |  | |  | |  | |  | |  | |  | |  | |  | |  | |  | |  | |  | |  | |  |  |  |  |  |  |  |  |  |  |  |  |  |  |  |  |  |  |  |  |  |  |  |  |  |  |  |  |  |  |  |  |

**Search string for PubMed:**

(("Renal Insufficiency, Chronic"[majr] OR "chronic kidney disease"[ti] OR "chronic kidney diseases"[ti] OR "chronic renal disease"[ti] OR "chronic renal diseases"[ti] OR "chronic kidney"[ti] OR "chronic renal"[ti] OR "CKD"[ti] OR "Kidney Failure, Chronic"[majr] OR "Chronic Kidney Failure"[ti] OR "Chronic Renal Failure"[ti] OR "end stage renal disease"[ti] OR "end stage renal diseases"[ti] OR "end stage renal"[ti] OR "endstage renal disease"[ti] OR "endstage renal"[ti] OR "end stage kidney disease"[ti] OR "end stage kidney diseases"[ti] OR "end stage kidney"[ti] OR "endstage kidney disease"[ti] OR "endstage kidney"[ti] OR "ESRD"[ti] OR "ESKD"[ti] OR ((end stage*[ti] OR endstage*[ti]) AND ("renal"[ti] OR kidney*[ti]))) AND ("Patient Preference"[majr] OR "preference"[ti] OR preferenc*[ti] OR "Patient Satisfaction"[majr] OR "satisfaction"[ti] OR satisfact*[ti] OR "Attitude to Health"[majr] OR "Health Knowledge, Attitudes, Practice"[majr] OR "Attitude"[majr] OR Opinion*[ti] OR "Communication"[majr:noexp] OR "Access to Information"[majr] OR "Communication Barriers"[majr] OR "Health Communication"[majr] OR "Information Literacy"[majr] OR "Health Literacy"[majr] OR "Information Seeking Behavior"[majr] OR "Negotiating"[majr] OR "Nonverbal Communication"[majr] OR "Persuasive Communication"[majr] OR Communicat*[ti] OR "Advance Care Planning"[majr] OR "Advance care planning"[ti] OR (("Interviews as Topic"[majr] OR Interview*[ti]) AND ("Patients"[Majr] OR "Patient"[ti] OR "Patients"[ti])) OR "Emotions"[majr] OR "Patient Education as Topic"[majr] OR (Experience*[ti] AND ("Patients"[Majr] OR "Patient"[ti] OR "Patients"[ti])) OR patient experienc*[ti] OR patients experienc*[ti] OR patient perspectiv*[ti] OR patients perspectiv*[ti] OR patients' perspectiv*[ti] OR ((futur*[ti] OR "Forecasting"[majr] OR forecast*[ti]) AND ("patient"[ti] OR "patients"[ti])) OR "Qualitative Research"[majr] OR "qualitative"[ti] OR qualitative*[ti] OR "Perception"[majr:noexp] OR "perception"[ti] OR "Adaptation, Psychological"[majr] OR "coping"[ti] OR "cope"[ti] OR "barrier"[ti] OR "barriers"[ti] OR "Focus Groups"[majr] OR "focus group"[ti] OR "focus groups"[ti] OR (("Qualitative Research"[mesh] OR "qualitative"[tw] OR qualitative*[tw]) AND ("Patient Preference"[mesh] OR "preference"[tw] OR preferenc*[tw] OR "Patient Satisfaction"[mesh] OR "satisfaction"[tw] OR satisfact*[tw] OR "Attitude to Health"[mesh] OR "Health Knowledge, Attitudes, Practice"[mesh] OR "Attitude"[mesh] OR Opinion*[tw] OR "Communication"[mesh:noexp] OR "Access to Information"[mesh] OR "Communication Barriers"[mesh] OR "Health Communication"[mesh] OR "Information Literacy"[mesh] OR "Health Literacy"[mesh] OR "Information Seeking Behavior"[mesh] OR "Negotiating"[mesh] OR "Nonverbal Communication"[mesh] OR "Persuasive Communication"[mesh] OR Communicat*[tw] OR "Advance Care Planning"[mesh] OR "Advance care planning"[tw] OR (("Interviews as Topic"[mesh] OR Interview*[tw]) AND ("Patients"[mesh] OR "Patient"[tw] OR "Patients"[tw])) OR "Emotions"[mesh] OR "Patient Education as Topic"[mesh] OR (Experience*[tw] AND ("Patients"[mesh] OR "Patient"[tw] OR "Patients"[tw])) OR patient experienc*[tw] OR patients experienc*[tw] OR patient perspectiv*[tw] OR patients perspectiv*[tw] OR patients' perspectiv*[tw] OR ((futur*[tw] OR "Forecasting"[mesh] OR forecast*[tw]) AND ("patient"[tw] OR "patients"[tw]))))) NOT ("Case Reports"[ptyp] OR "Evaluation Studies"[ptyp] OR "Scientific Integrity Review"[ptyp] OR "Twin Study"[ptyp]) NOT ("Animals"[mesh] NOT "Humans"[mesh]) AND (english[la] OR dutch[la]))

**Search string for Embase:**

((exp *"chronic kidney failure"/ OR "chronic kidney disease".ti OR "chronic kidney diseases".ti OR "chronic renal disease".ti OR "chronic renal diseases".ti OR "chronic kidney".ti OR "chronic renal".ti OR "CKD".ti OR "Chronic Kidney Failure".ti OR "Chronic Renal Failure".ti OR *"end stage renal disease"/ OR "end stage renal disease".ti OR "end stage renal diseases".ti OR "end stage renal".ti OR "endstage renal disease".ti OR "endstage renal".ti OR "end stage kidney disease".ti OR "end stage kidney diseases".ti OR "end stage kidney".ti OR "endstage kidney disease".ti OR "endstage kidney".ti OR "ESRD".ti OR "ESKD".ti OR ((end stage*.ti OR endstage*.ti) AND ("renal".ti OR kidney*.ti))) AND (*"Patient Preference"/ OR "preference".ti OR preferenc*.ti OR *"Patient Satisfaction"/ OR "satisfaction".ti OR satisfact*.ti OR exp *"Patient Attitude"/ OR *"Attitude to Health"/ OR exp *"Attitude"/ OR Opinion*.ti OR *"Interpersonal Communication"/ OR exp *"Access to Information"/ OR *"Communication Barrier"/ OR *"Medical Information"/ OR *"Information Literacy"/ OR *"Health Literacy"/ OR *"Information Seeking"/ OR exp *"Nonverbal Communication"/ OR *"Persuasive Communication"/ OR Communicat*.ti OR *"Advance Care Planning"/ OR "Advance care planning".ti OR ((exp *"Interview"/ OR Interview*.ti) AND (exp *"Patient"/ OR "patient".ti OR "patients".ti)) OR exp *"Emotion"/ OR *"Patient Education"/ OR (Experience*.ti AND (exp *"Patient"/ OR "Patient".ti OR "Patients".ti)) OR patient experienc*.ti OR patients experienc*.ti OR patient perspectiv*.ti OR patients perspectiv*.ti OR patients' perspectiv*.ti OR ((futur*.ti OR *"Forecasting"/ OR forecast*.ti) AND ("patient".ti OR "patients".ti)) OR exp *"Qualitative Research"/ OR "qualitative".ti OR qualitative*.ti OR *"Perception"/ OR "perception".ti OR exp *"Coping Behavior"/ OR "coping".ti OR "cope".ti OR "barrier".ti OR "barriers".ti OR "focus group".ti OR "focus groups".ti OR ((exp "Qualitative Research"/ OR "qualitative".mp OR qualitative*.mp) AND ("Patient Preference"/ OR "preference".mp OR preferenc*.mp OR "Patient Satisfaction"/ OR "satisfaction".mp OR satisfact*.mp OR exp "Patient Attitude"/ OR "Attitude to Health"/ OR exp "Attitude"/ OR Opinion*.mp OR "Interpersonal Communication"/ OR exp "Access to Information"/ OR "Communication Barrier"/ OR "Medical Information"/ OR "Information Literacy"/ OR "Health Literacy"/ OR "Information Seeking"/ OR exp "Nonverbal Communication"/ OR "Persuasive Communication"/ OR Communicat*.mp OR "Advance Care Planning"/ OR "Advance care planning".mp OR ((exp "Interview"/ OR Interview*.mp) AND (exp "Patient"/ OR "patient".mp OR "patients".mp)) OR exp "Emotion"/ OR "Patient Education"/ OR (Experience*.mp AND (exp "Patient"/ OR "Patient".mp OR "Patients".mp)) OR patient experienc*.mp OR patients experienc*.mp OR patient perspectiv*.mp OR patients perspectiv*.mp OR patients' perspectiv*.mp OR ((futur*.mp OR "Forecasting"/ OR forecast*.mp) AND ("patient".mp OR "patients".mp))))) NOT ("Case Report"/ OR "Evaluation Study"/ OR exp "Twin Study"/) AND exp "Humans"/ AND (english.la OR dutch.la))

**Search string for Web of Science:**

(TI=("chronic kidney failure" OR "chronic kidney disease" OR "chronic kidney diseases" OR "chronic renal disease" OR "chronic renal diseases" OR "chronic kidney" OR "chronic renal" OR "CKD" OR "Chronic Kidney Failure" OR "Chronic Renal Failure" OR "end stage renal disease" OR "end stage renal disease" OR "end stage renal diseases" OR "end stage renal" OR "endstage renal disease" OR "endstage renal" OR "end stage kidney disease" OR "end stage kidney diseases" OR "end stage kidney" OR "endstage kidney disease" OR "endstage kidney" OR "ESRD" OR "ESKD" OR ((end stage* OR endstage*) AND ("renal" OR kidney*))) AND (TI=("Patient Preference" OR "preference" OR preferenc* OR "Patient Satisfaction" OR "satisfaction" OR satisfact* OR "Patient Attitude" OR "Attitude to Health" OR "Attitude" OR Opinion* OR "Interpersonal Communication" OR "Access to Information" OR "Communication Barrier" OR "Medical Information" OR "Information Literacy" OR "Health Literacy" OR "Information Seeking" OR "Nonverbal Communication" OR "Persuasive Communication" OR Communicat* OR "Advance Care Planning" OR "Advance care planning" OR (("Interview" OR Interview*) AND ("Patient" OR "patient" OR "patients")) OR "Emotion" OR "Patient Education" OR (Experience* AND ("Patient" OR "Patient" OR "Patients")) OR patient experienc* OR patients experienc* OR patient perspectiv* OR patients perspectiv* OR patients' perspectiv* OR ((futur* OR "Forecasting" OR forecast*) AND ("patient" OR "patients")) OR "Qualitative Research" OR "qualitative" OR qualitative* OR "perception" OR "coping" OR "cope" OR "barrier" OR "barriers" OR "focus group" OR "focus groups") OR TS=(("Qualitative Research" OR "qualitative" OR qualitative*) AND ("Patient Preference" OR "preference" OR preferenc* OR "Patient Satisfaction" OR "satisfaction" OR satisfact* OR "Patient Attitude" OR "Attitude to Health" OR "Attitude" OR Opinion* OR "Interpersonal Communication" OR "Access to Information" OR "Communication Barrier" OR "Medical Information" OR "Information Literacy" OR "Health Literacy" OR "Information Seeking" OR "Nonverbal Communication" OR "Persuasive Communication" OR Communicat* OR "Advance Care Planning" OR "Advance care planning" OR (("Interview" OR Interview*) AND ("Patient" OR "patient" OR "patients")) OR "Emotion" OR "Patient Education" OR (Experience* AND ("Patient" OR "Patient" OR "Patients")) OR patient experienc* OR patients experienc* OR patient perspectiv* OR patients perspectiv* OR patients' perspectiv* OR ((futur* OR "Forecasting" OR forecast*) AND ("patient" OR "patients")))))) NOT TI=("Case Report" OR "Evaluation Study" OR "Twin Study") AND la=(english OR dutch) NOT ti=(veterinary OR rabbit OR rabbits OR animal OR animals OR mouse OR mice OR rodent OR rodents OR rat OR rats OR pig OR pigs OR porcine OR horse* OR equine OR cow OR cows OR bovine OR goat OR goats OR sheep OR ovine OR canine OR dog OR dogs OR feline OR cat OR cats)

**Search string for Cochrane Library:**

(("chronic kidney failure" OR "chronic kidney disease" OR "chronic kidney diseases" OR "chronic renal disease" OR "chronic renal diseases" OR "chronic kidney" OR "chronic renal" OR "CKD" OR "Chronic Kidney Failure" OR "Chronic Renal Failure" OR "end stage renal disease" OR "end stage renal disease" OR "end stage renal diseases" OR "end stage renal" OR "endstage renal disease" OR "endstage renal" OR "end stage kidney disease" OR "end stage kidney diseases" OR "end stage kidney" OR "endstage kidney disease" OR "endstage kidney" OR "ESRD" OR "ESKD" OR ((end stage* OR endstage*) AND ("renal" OR kidney*))) AND ("Patient Preference" OR "preference" OR preferenc* OR "Patient Satisfaction" OR "satisfaction" OR satisfact* OR "Patient Attitude" OR "Attitude to Health" OR "Attitude" OR Opinion* OR "Interpersonal Communication" OR "Access to Information" OR "Communication Barrier" OR "Medical Information" OR "Information Literacy" OR "Health Literacy" OR "Information Seeking" OR "Nonverbal Communication" OR "Persuasive Communication" OR Communicat* OR "Advance Care Planning" OR "Advance care planning" OR (("Interview" OR Interview*) AND ("Patient" OR "patient" OR "patients")) OR "Emotion" OR "Patient Education" OR (Experience* AND ("Patient" OR "Patient" OR "Patients")) OR patient experienc* OR patients experienc* OR patient perspectiv* OR patients perspectiv* OR patients' perspectiv* OR ((futur* OR "Forecasting" OR forecast*) AND ("patient" OR "patients")) OR "Qualitative Research" OR "qualitative" OR qualitative* OR "perception" OR "coping" OR "cope" OR "barrier" OR "barriers" OR "focus group" OR "focus groups")):ti OR (("chronic kidney failure" OR "chronic kidney disease" OR "chronic kidney diseases" OR "chronic renal disease" OR "chronic renal diseases" OR "chronic kidney" OR "chronic renal" OR "CKD" OR "Chronic Kidney Failure" OR "Chronic Renal Failure" OR "end stage renal disease" OR "end stage renal disease" OR "end stage renal diseases" OR "end stage renal" OR "endstage renal disease" OR "endstage renal" OR "end stage kidney disease" OR "end stage kidney diseases" OR "end stage kidney" OR "endstage kidney disease" OR "endstage kidney" OR "ESRD" OR "ESKD" OR ((end stage* OR endstage*) AND ("renal" OR kidney*))):ti AND (("Qualitative Research" OR "qualitative" OR qualitative*) AND ("Patient Preference" OR "preference" OR preferenc* OR "Patient Satisfaction" OR "satisfaction" OR satisfact* OR "Patient Attitude" OR "Attitude to Health" OR "Attitude" OR Opinion* OR "Interpersonal Communication" OR "Access to Information" OR "Communication Barrier" OR "Medical Information" OR "Information Literacy" OR "Health Literacy" OR "Information Seeking" OR "Nonverbal Communication" OR "Persuasive Communication" OR Communicat* OR "Advance Care Planning" OR "Advance care planning" OR (("Interview" OR Interview*) AND ("Patient" OR "patient" OR "patients")) OR "Emotion" OR "Patient Education" OR (Experience* AND ("Patient" OR "Patient" OR "Patients")) OR patient experienc* OR patients experienc* OR patient perspectiv* OR patients perspectiv* OR patients' perspectiv* OR ((futur* OR "Forecasting" OR forecast*) AND ("patient" OR "patients")))):ti,ab,kw)

**Search string for PsycINFO:**

TI(("chronic kidney failure" OR "chronic kidney disease" OR "chronic kidney diseases" OR "chronic renal disease" OR "chronic renal diseases" OR "chronic kidney" OR "chronic renal" OR "CKD" OR "Chronic Kidney Failure" OR "Chronic Renal Failure" OR "end stage renal disease" OR "end stage renal disease" OR "end stage renal diseases" OR "end stage renal" OR "endstage renal disease" OR "endstage renal" OR "end stage kidney disease" OR "end stage kidney diseases" OR "end stage kidney" OR "endstage kidney disease" OR "endstage kidney" OR "ESRD" OR "ESKD" OR ((end stage* OR endstage*) AND ("renal" OR kidney*))) AND ("Patient Preference" OR "preference" OR preferenc* OR "Patient Satisfaction" OR "satisfaction" OR satisfact* OR "Patient Attitude" OR "Attitude to Health" OR "Attitude" OR Opinion* OR "Interpersonal Communication" OR "Access to Information" OR "Communication Barrier" OR "Medical Information" OR "Information Literacy" OR "Health Literacy" OR "Information Seeking" OR "Nonverbal Communication" OR "Persuasive Communication" OR Communicat* OR "Advance Care Planning" OR "Advance care planning" OR (("Interview" OR Interview*) AND ("Patient" OR "patient" OR "patients")) OR "Emotion" OR "Patient Education" OR (Experience* AND ("Patient" OR "Patient" OR "Patients")) OR patient experienc* OR patients experienc* OR patient perspectiv* OR patients perspectiv* OR patients' perspectiv* OR ((futur* OR "Forecasting" OR forecast*) AND ("patient" OR "patients")) OR "Qualitative Research" OR "qualitative" OR qualitative* OR "perception" OR "coping" OR "cope" OR "barrier" OR "barriers" OR "focus group" OR "focus groups"))

**Search string for Emcare:**

((exp *"chronic kidney failure"/ OR "chronic kidney disease".ti OR "chronic kidney diseases".ti OR "chronic renal disease".ti OR "chronic renal diseases".ti OR "chronic kidney".ti OR "chronic renal".ti OR "CKD".ti OR "Chronic Kidney Failure".ti OR "Chronic Renal Failure".ti OR *"end stage renal disease"/ OR "end stage renal disease".ti OR "end stage renal diseases".ti OR "end stage renal".ti OR "endstage renal disease".ti OR "endstage renal".ti OR "end stage kidney disease".ti OR "end stage kidney diseases".ti OR "end stage kidney".ti OR "endstage kidney disease".ti OR "endstage kidney".ti OR "ESRD".ti OR "ESKD".ti OR ((end stage*.ti OR endstage*.ti) AND ("renal".ti OR kidney*.ti))) AND (*"Patient Preference"/ OR "preference".ti OR preferenc*.ti OR *"Patient Satisfaction"/ OR "satisfaction".ti OR satisfact*.ti OR exp *"Patient Attitude"/ OR *"Attitude to Health"/ OR exp *"Attitude"/ OR Opinion*.ti OR *"Interpersonal Communication"/ OR exp *"Access to Information"/ OR *"Communication Barrier"/ OR *"Medical Information"/ OR *"Information Literacy"/ OR *"Health Literacy"/ OR *"Information Seeking"/ OR exp *"Nonverbal Communication"/ OR *"Persuasive Communication"/ OR Communicat*.ti OR *"Advance Care Planning"/ OR "Advance care planning".ti OR ((exp *"Interview"/ OR Interview*.ti) AND (exp *"Patient"/ OR "patient".ti OR "patients".ti)) OR exp *"Emotion"/ OR *"Patient Education"/ OR (Experience*.ti AND (exp *"Patient"/ OR "Patient".ti OR "Patients".ti)) OR patient experienc*.ti OR patients experienc*.ti OR patient perspectiv*.ti OR patients perspectiv*.ti OR patients' perspectiv*.ti OR ((futur*.ti OR *"Forecasting"/ OR forecast*.ti) AND ("patient".ti OR "patients".ti)) OR exp *"Qualitative Research"/ OR "qualitative".ti OR qualitative*.ti OR *"Perception"/ OR "perception".ti OR exp *"Coping Behavior"/ OR "coping".ti OR "cope".ti OR "barrier".ti OR "barriers".ti OR "focus group".ti OR "focus groups".ti OR ((exp "Qualitative Research"/ OR "qualitative".mp OR qualitative*.mp) AND ("Patient Preference"/ OR "preference".mp OR preferenc*.mp OR "Patient Satisfaction"/ OR "satisfaction".mp OR satisfact*.mp OR exp "Patient Attitude"/ OR "Attitude to Health"/ OR exp "Attitude"/ OR Opinion*.mp OR "Interpersonal Communication"/ OR exp "Access to Information"/ OR "Communication Barrier"/ OR "Medical Information"/ OR "Information Literacy"/ OR "Health Literacy"/ OR "Information Seeking"/ OR exp "Nonverbal Communication"/ OR "Persuasive Communication"/ OR Communicat*.mp OR "Advance Care Planning"/ OR "Advance care planning".mp OR ((exp "Interview"/ OR Interview*.mp) AND (exp "Patient"/ OR "patient".mp OR "patients".mp)) OR exp "Emotion"/ OR "Patient Education"/ OR (Experience*.mp AND (exp "Patient"/ OR "Patient".mp OR "Patients".mp)) OR patient experienc*.mp OR patients experienc*.mp OR patient perspectiv*.mp OR patients perspectiv*.mp OR patients' perspectiv*.mp OR ((futur*.mp OR "Forecasting"/ OR forecast*.mp) AND ("patient".mp OR "patients".mp))))) NOT ("Case Report"/ OR "Evaluation Study"/ OR exp "Twin Study"/) AND exp "Humans"/ AND (english.la OR dutch.la))

1. Beard BH. Fear of death and fear of life. The dilemma in chronic renal failure, hemodialysis, and kidney transplantation. *Archives of general psychiatry* 1969;21(3):373-80. [published Online First: 1969/09/01]

2. Tong A, Sainsbury P, Carter SM, et al. Patients' priorities for health research: focus group study of patients with chronic kidney disease. *Nephrology, dialysis, transplantation : official publication of the European Dialysis and Transplant Association - European Renal Association* 2008;23(10):3206-14. doi: 10.1093/ndt/gfn207 [published Online First: 2008/05/01]

3. Lee A, Gudex C, Povlsen JV, et al. Patients' views regarding choice of dialysis modality. *Nephrology, dialysis, transplantation : official publication of the European Dialysis and Transplant Association - European Renal Association* 2008;23(12):3953-9. doi: 10.1093/ndt/gfn365 [published Online First: 2008/07/01]

4. Tong A, Sainsbury P, Chadban S, et al. Patients' experiences and perspectives of living with CKD. *American journal of kidney diseases : the official journal of the National Kidney Foundation* 2009;53(4):689-700. doi: 10.1053/j.ajkd.2008.10.050 [published Online First: 2009/02/14]

5. Boulware LE, Hill-Briggs F, Kraus ES, et al. Identifying and addressing barriers to African American and non-African American families' discussions about preemptive living related kidney transplantation. *Progress in transplantation (Aliso Viejo, Calif)* 2011;21(2):97-104; quiz 05. [published Online First: 2011/07/09]

6. Morton RL, Tong A, Webster AC, et al. Characteristics of dialysis important to patients and family caregivers: a mixed methods approach. *Nephrology, dialysis, transplantation : official publication of the European Dialysis and Transplant Association - European Renal Association* 2011;26(12):4038-46. doi: 10.1093/ndt/gfr177 [published Online First: 2011/04/13]

7. Llewellyn H, Low J, Smith G, et al. Narratives of continuity among older people with late stage chronic kidney disease who decline dialysis. *Social science & medicine (1982)* 2014;114:49-56. doi: 10.1016/j.socscimed.2014.05.037 [published Online First: 2014/06/10]

8. Meuleman Y, Ten Brinke L, Kwakernaak AJ, et al. Perceived Barriers and Support Strategies for Reducing Sodium Intake in Patients with Chronic Kidney Disease: a Qualitative Study. *International journal of behavioral medicine* 2015;22(4):530-9. doi: 10.1007/s12529-014-9447-x [published Online First: 2014/10/10]

9. Lederer S, Fischer MJ, Gordon HS, et al. Barriers to effective communication between veterans with chronic kidney disease and their healthcare providers. *Clinical kidney journal* 2015;8(6):766-71. doi: 10.1093/ckj/sfv079 [published Online First: 2015/11/28]

10. Seah AS, Tan F, Srinivas S, et al. Opting out of dialysis - Exploring patients' decisions to forego dialysis in favour of conservative non-dialytic management for end-stage renal disease. *Health expectations : an international journal of public participation in health care and health policy* 2015;18(5):1018-29. doi: 10.1111/hex.12075 [published Online First: 2013/05/08]

11. Tong A, Brown MA, Winkelmayer WC, et al. Perspectives on Pregnancy in Women With CKD: A Semistructured Interview Study. *American journal of kidney diseases : the official journal of the National Kidney Foundation* 2015;66(6):951-61. doi: 10.1053/j.ajkd.2015.08.023 [published Online First: 2015/10/11]

12. Kang H, Stenfors-Hayes T. Feeling Well and Having Good Numbers: Renal Patients' Encounter With Clinical Uncertainties and the Responsibility to "Live Well". *Qualitative health research* 2016;26(12):1591-602. doi: 10.1177/1049732315591484 [published Online First: 2015/07/02]

13. Lederer S, Fischer MJ, Gordon HS, et al. A question prompt sheet for adult patients with chronic kidney disease. *BMC nephrology* 2016;17(1):155. doi: 10.1186/s12882-016-0362-z [published Online First: 2016/10/21]

14. Walker RC, Howard K, Morton RL, et al. Patient and caregiver values, beliefs and experiences when considering home dialysis as a treatment option: a semi-structured interview study. *Nephrology, dialysis, transplantation : official publication of the European Dialysis and Transplant Association - European Renal Association* 2016;31(1):133-41. doi: 10.1093/ndt/gfv330 [published Online First: 2015/09/09]

15. Lo C, Ilic D, Teede H, et al. The Perspectives of Patients on Health-Care for Co-Morbid Diabetes and Chronic Kidney Disease: A Qualitative Study. *PloS one* 2016;11(1):e0146615. doi: 10.1371/journal.pone.0146615 [published Online First: 2016/01/06]

16. Loiselle m-c, Michaud C, M. O'Connor A. Decisional Needs Assessment to Help Patients with Advanced Chronic Kidney Disease Make Better Dialysis Choices2016.

17. Morris RL, Ashcroft D, Phipps D, et al. Preventing Acute Kidney Injury: a qualitative study exploring 'sick day rules' implementation in primary care. *BMC family practice* 2016;17:91. doi: 10.1186/s12875-016-0480-5 [published Online First: 2016/07/28]

18. Subramanian L, Quinn M, Zhao J, et al. Coping with kidney disease - qualitative findings from the Empowering Patients on Choices for Renal Replacement Therapy (EPOCH-RRT) study. *BMC nephrology* 2017;18(1):119. doi: 10.1186/s12882-017-0542-5 [published Online First: 2017/04/05]

19. Walker RC, Walker S, Morton RL, et al. Maori patients' experiences and perspectives of chronic kidney disease: a New Zealand qualitative interview study. *BMJ open* 2017;7(1):e013829. doi: 10.1136/bmjopen-2016-013829 [published Online First: 2017/01/21]

20. Smekal MD, Tam-Tham H, Finlay J, et al. Perceived Benefits and Challenges of a Risk-Based Approach to Multidisciplinary Chronic Kidney Disease Care: A Qualitative Descriptive Study. *Canadian journal of kidney health and disease* 2018;5:2054358118763809. doi: 10.1177/2054358118763809 [published Online First: 2018/04/11]

21. Andrew J. The pre-dialysis experience--are individual needs being met? *EDTNA/ERCA journal (English ed)* 2001;27(2):72-4. [published Online First: 2002/03/01]

22. Iles-Smith H. Perceptions and experiences of pre-dialysis patients. *EDTNA/ERCA journal (English ed)* 2005;31(3):130-3. [published Online First: 2005/12/21]

23. Tweed AE, Ceaser K. Renal replacement therapy choices for pre-dialysis renal patients. *British journal of nursing (Mark Allen Publishing)* 2005;14(12):659-64. doi: 10.12968/bjon.2005.14.12.18287 [published Online First: 2005/07/13]

24. Costantini L, Beanlands H, McCay E, et al. The self-management experience of people with mild to moderate chronic kidney disease. *Nephrology nursing journal : journal of the American Nephrology Nurses' Association* 2008;35(2):147-55; quiz 56. [published Online First: 2008/05/14]

25. Sakraida TJ, Robinson MV. Health literacy self-management by patients with type 2 diabetes and stage 3 chronic kidney disease. *Western journal of nursing research* 2009;31(5):627-47. doi: 10.1177/0193945909334096 [published Online First: 2009/04/25]

26. Noble H, Meyer PJ, Bridge DJ, et al. Exploring symptoms in patients managed without dialysis: a qualitative research study. *Journal of renal care* 2010;36(1):9-15. doi: 10.1111/j.1755-6686.2010.00140.x [published Online First: 2010/03/11]

27. de Brito-Ashurst I, Perry L, Sanders TA, et al. Barriers and facilitators of dietary sodium restriction amongst Bangladeshi chronic kidney disease patients. *Journal of human nutrition and dietetics : the official journal of the British Dietetic Association* 2011;24(1):86-95. doi: 10.1111/j.1365-277X.2010.01129.x [published Online First: 2010/12/01]

28. Nygardh A, Malm D, Wikby K, et al. The experience of empowerment in the patient-staff encounter: the patient's perspective. *Journal of clinical nursing* 2012;21(5-6):897-904. doi: 10.1111/j.1365-2702.2011.03901.x [published Online First: 2011/11/16]

29. Sakraida TJ, Robinson MV. Mental health and relational self-management experiences of patients with type 2 diabetes and stage 3 chronic kidney disease. *Issues in mental health nursing* 2012;33(11):786-96. doi: 10.3109/01612840.2012.713446 [published Online First: 2012/11/14]

30. Walker R, James H, Burns A. Adhering to behaviour change in older pre-dialysis populations--what do patients think? A qualitative study. *Journal of renal care* 2012;38(1):34-42. doi: 10.1111/j.1755-6686.2012.00262.x [published Online First: 2012/03/01]

31. Johnston S, Noble H. Factors influencing patients with stage 5 chronic kidney disease to opt for conservative management: a practitioner research study. *Journal of clinical nursing* 2012;21(9-10):1215-22. doi: 10.1111/j.1365-2702.2011.04001.x [published Online First: 2012/03/06]

32. McKillop G, Joy J. Patients' experience and perceptions of polypharmacy in chronic kidney disease and its impact on adherent behaviour. *Journal of renal care* 2013;39(4):200-7. doi: 10.1111/j.1755-6686.2013.12037.x [published Online First: 2013/11/20]

33. Lin CC, Chen MC, Hsieh HF, et al. Illness representations and coping processes of Taiwanese patients with early-stage chronic kidney disease. *The journal of nursing research : JNR* 2013;21(2):120-8. doi: 10.1097/jnr.0b013e3182921fb8 [published Online First: 2013/05/18]

34. Lopez-Vargas PA, Tong A, Phoon RK, et al. Knowledge deficit of patients with stage 1-4 CKD: a focus group study. *Nephrology (Carlton, Vic)* 2014;19(4):234-43. doi: 10.1111/nep.12206 [published Online First: 2014/01/17]

35. Tangkiatkumjai M, Boardman H, Praditpornsilpa K, et al. Reasons why Thai patients with chronic kidney disease use or do not use herbal and dietary supplements. *BMC complementary and alternative medicine* 2014;14:473. doi: 10.1186/1472-6882-14-473 [published Online First: 2014/12/08]

36. Clarke AL, Young HM, Hull KL, et al. Motivations and barriers to exercise in chronic kidney disease: a qualitative study. *Nephrology, dialysis, transplantation : official publication of the European Dialysis and Transplant Association - European Renal Association* 2015;30(11):1885-92. doi: 10.1093/ndt/gfv208 [published Online First: 2015/06/10]

37. Erlang AS, Nielsen IH, Hansen HO, et al. PATIENTS EXPERIENCES OF INVOLVEMENT IN CHOICE OF DIALYSIS MODE. *Journal of renal care* 2015;41(4):260-7. doi: 10.1111/jorc.12141 [published Online First: 2015/09/30]

38. Shirazian S, Crnosija N, Weinger K, et al. The self-management experience of patients with type 2 diabetes and chronic kidney disease: A qualitative study. *Chronic illness* 2016;12(1):18-28. doi: 10.1177/1742395315614381 [published Online First: 2015/11/06]

39. Wright Nunes J, Roney M, Kerr E, et al. A diagnosis of chronic kidney disease: despite fears patients want to know early. *Clinical nephrology* 2016;86(2):78-86. doi: 10.5414/cn108831 [published Online First: 2016/06/28]

40. Wu CC, Lin CC, Hsieh HF, et al. Lived experiences and illness representation of Taiwanese patients with late-stage chronic kidney disease. *Journal of health psychology* 2016;21(12):2788-98. doi: 10.1177/1359105315587134 [published Online First: 2015/06/11]

41. Schipper K, van der Borg WE, de Jong-Camerik J, et al. Living with moderate to severe renal failure from the perspective of patients. *BMC nephrology* 2016;17(1):48. doi: 10.1186/s12882-016-0263-1 [published Online First: 2016/05/18]

42. Bowling CB, Vandenberg AE, Phillips LS, et al. Older Patients' Perspectives on Managing Complexity in CKD Self-Management. *Clinical journal of the American Society of Nephrology : CJASN* 2017;12(4):635-43. doi: 10.2215/cjn.06850616 [published Online First: 2017/04/09]

43. Havas K, Douglas C, Bonner A. Closing the loop in person-centered care: patient experiences of a chronic kidney disease self-management intervention. *Patient preference and adherence* 2017;11:1963-73. doi: 10.2147/ppa.s147831 [published Online First: 2017/12/15]

44. Lovell S, Walker RJ, Schollum JB, et al. To dialyse or delay: a qualitative study of older New Zealanders' perceptions and experiences of decision-making, with stage 5 chronic kidney disease. *BMJ open* 2017;7(3):e014781. doi: 10.1136/bmjopen-2016-014781 [published Online First: 2017/04/01]

45. Pugh-Clarke K, Read SC, Sim J. Symptom experience in non-dialysis-dependent chronic kidney disease: A qualitative descriptive study. *Journal of renal care* 2017;43(4):197-208. doi: 10.1111/jorc.12208 [published Online First: 2017/06/15]

46. Campbell-Crofts S, Stewart G. How perceived feelings of "wellness" influence the decision-making of people with predialysis chronic kidney disease. *Journal of clinical nursing* 2018;27(7-8):1561-71. doi: 10.1111/jocn.14220 [published Online First: 2017/12/15]
